# Unveiling the mitophagy puzzle in non-alcoholic fatty liver disease (NAFLD): Six hub genes for early diagnosis and immune modulatory roles

**DOI:** 10.1016/j.heliyon.2024.e28935

**Published:** 2024-03-31

**Authors:** Zhenguo Luo, Shu Yan, Yu Chao, Ming Shen

**Affiliations:** a Department of Internal Medicine, The First People's Hospital of Changzhou, The Third Affiliated Hospital of Soochow University, Changzhou, Jiangsu, China; b Department of Gastroenterology, The First People's Hospital of Changzhou, The Third Affiliated Hospital of Soochow University, Changzhou, Jiangsu, China; c Department of Cardiology, The 926th Hospital of the Joint Logistic Support Force of PLA, Affiliated Hospital of Kunming University of Science and Technology, Kaiyuan, Yunnan, China

**Keywords:** Non-alcoholic fatty liver disease, Mitophagy, Diagnostic model, Immune infiltration, Bioinformatics analysis

## Abstract

**Background:**

Non-alcoholic fatty liver disease (NAFLD) stands as a predominant chronic liver ailment globally, yet its pathogenesis remains elusive. This study aims to identify Hub mitophagy-related genes (MRGs), and explore the underlying pathological mechanisms through which these hub genes regulate NAFLD.

**Methods:**

A total of 3 datasets were acquired from the GEO database and integrated to identify differentially expressed genes (DEGs) in NAFLD and perform Gene Set Enrichment Analysis (GSEA). By intersecting DEGs with MRGs, mitophagy-related differentially expressed genes (MRDEGs) were obtained. Then, hub MRGs with diagnostic biomarker capability for NAFLD were screened and a diagnostic prediction model was constructed and assessed using Nomogram, Decision Curve Analysis (DCA), and ROC curves. Functional enrichment analysis was conducted on the identified hub genes to explore their biological significance. Additionally, regulatory networks were constructed using databases. NAFLD was stratified into high and low-risk groups based on the Riskscore from the diagnostic prediction model. Furthermore, single-sample gene set enrichment analysis (ssGSEA) and CIBERSORT algorithms were employed to analyze immune cell infiltration patterns and the relationship between Hub MRGs and immune cells.

**Results:**

The integrated dataset comprised 122 NAFLD samples and 31 control samples. After screening, 18 MRDEGs were identified. Subsequently, six hub MRGs (NR4A1, PPP2R2A, P4HA1, TUBB6, DUSP1, NAMPT) with diagnostic potential were selected through WGCNA, logistic regression, SVM, RF, and LASSO models, all significantly downregulated in NAFLD samples compared to the control group. A diagnostic prediction model based on these six genes demonstrated robust predictive performance. Functional enrichment analysis of the six hub genes revealed involvement in processes such as protein phosphorylation or dephosphorylation. Correlation analysis demonstrated a significant association between hub MRGs and infiltrating immune cells.

**Conclusion:**

We identified six hub MRGs in NAFLD and constructed a diagnostic prediction model based on these six genes, applicable for early NAFLD diagnosis. These genes may participate in regulating NAFLD progression through the modulation of mitophagy and immune activation. Our findings may contribute to subsequent clinical and basic research on NAFLD.

## 1 Introduction

Nonalcoholic fatty liver disease (NAFLD) is a metabolic syndrome characterized by the excessive accumulation of fat in the liver. In its early stages, it manifests as nonalcoholic fatty liver (NAFL) and nonalcoholic steatohepatitis (NASH), with some patients progressing to liver cirrhosis and hepatocellular carcinoma due to fibrosis. Epidemiological studies indicate that from 2015 to 2021, the overall prevalence of NAFLD increased from 25.24% to 29.38%, with approximately 10%–29% of NASH patients developing liver cirrhosis within a decade [1]. NAFLD is emerging as a major global chronic liver disease, posing a substantial health threat to nearly 1.7 billion people worldwide and imposing significant burdens on individuals, families, and healthcare systems [2]. The mechanistic underpinnings of NAFLD remain incompletely elucidated, leading to a lack of reliable early non-invasive diagnostic tools, treatment strategies, and medications [3]. Investigating the pathogenic mechanisms of NAFLD, identifying specific biomarkers and constructing diagnostic prediction models, also serve as effective therapeutic targets for the early diagnosis, prevention and treatment of NAFLD, bearing crucial clinical significance.

In recent decades, research into the molecular mechanisms underlying NAFLD has progressed from the early "two-hit theory" to the current "multiple-hit theory", including lipid metabolism abnormalities, oxidative damage, innate immunity, cytokine release, gut-liver axis dysfunction, insulin resistance, and genetic factors, etc [4]. Studies indicated frequent mitochondrial dysfunction in the livers of NAFLD patients, characterized by structural abnormalities, oxidative stress, and disruption of the mitochondrial respiratory chain. These mitochondrial abnormalities exacerbated energy metabolism disruption, increased oxidative stress, and cellular damage, thereby intensifying the pathological progression of NAFLD[5]. Mitochondrial autophagy, or mitophagy, is a selective autophagic process that specifically clears damaged mitochondria. It serves as a critical mechanism for mitochondrial quality control, regulating cellular mitochondrial quantity to maintain mitochondrial homeostasis and normal functioning [6]. Recent reports have confirmed the vital role of mitophagy in clearing damaged mitochondria in NAFLD patients. A study in mice revealed that deficiencies in PTEN-induced putative kinase 1(PINK1) or Parkin led to a loss of mitophagy, exacerbating mitochondrial dysfunction and thereby accelerating the progression of NAFLD [7]. Generally, mitophagy is perceived as a safeguard mechanism during the extended development of NAFLD[8]. Certain small molecule compounds have demonstrated potential in mitigating NAFLD by enhancing mitophagy [9,10]. Exploring the regulatory mechanisms of mitophagy in NAFLD and intervening effectively in mitophagy shows great potential for preventing NAFLD progression. However, the association and role of mitophagy-related genes (MRGs) in NAFLD remain unexplored.

Additionally, recent studies indicate that mitophagy regulates the immune microenvironment in NAFLD progression. The intricate interplay between hepatic cells and the immune system constitutes a crucial pathway in the onset and progression of NAFLD. In NAFLD, the activation of immune cells can instigate inflammation and liver damage [11]. Mitophagy, pivotal in governing the secretion of inflammatory cytokines and maintaining the homeostasis and differentiation of immune cells, is intricately linked to the pathogenesis of immune and inflammatory disorders [12,13]. A study delineated that the impairment of mitophagy promotes cytoplasmic leakage of macrophage self-mtDNA, leading to macrophage STING activation and subsequent hepatic inflammation and fibrosis [14]. However, the reciprocal interaction between mitophagy and immune infiltration in NAFLD progression remains elusive, necessitating further investigation.

This study utilized the GEO database to analyze differentially expressed genes (DEGs) in NAFLD and Mitophagy-related differentially expressed genes (MRDEGs). Through weighted gene co-expression network analysis (WGCNA), we identified gene modules highly correlated with NAFLD and screened MRDEGs. A diagnostic model was initially constructed using logistic regression. Subsequently, we employed various machine learning algorithms includingSupport Vector Machine (SVM), Random Forest (RF), and Least Absolute Shrinkage and Selection Operator (LASSO) models to select Hub MRGs with biomarker capability for NAFLD. And a diagnostic prediction model was finally constructed. The diagnostic prediction model was assessed using nomogram, decision curve analysis (DCA) plots, and receiver operating characteristic (ROC) curves. Subsequent functional enrichment analyses were conducted to better understand the biological significance of these hub genes. Meanwhile, the regulatory networks of transcription factors (TFs), RNA-binding proteins (RBPs), microRNAs (miRNAs), and potential drugs or small molecules for the hub genes were constructed, offering a reference for future studies on the regulatory mechanisms of these hub genes. Based on single-sample gene set enrichment analysis (ssGSEA) and the CIBERSORT algorithm, we analyzed differences in immune cell infiltration between high and low-risk group of NAFLD and their connection with hub genes. This study comprehensively elucidates the role of mitophagy in NAFLD by integrating various analytical methods, including gene expression, functional enrichment, and immune infiltration analyses. Our study findings may potentially impact clinical practice by providing biomarker-capable Mitophagy-related genes for the diagnosis and treatment of NAFLD, as well as deepen the understanding of the pathological mechanisms of NAFLD, serving as valuable reference for future clinical practice and research endeavors.

## 2 Materials and methods

### 2.1 Data acquisition and pretreatment

A total of 3 NAFLD-related datasets GSE49541[15], GSE89632[16] and GSE63067[17] were retrieved from the Gene Expression Omnibus (GEO) database [18] using the R package GEOquery [19]. All three datasets originated from Homo sapiens. The platform of GSE89632 dataset was GPL14951(Illumina HumanHT-12 WG-DASL V4.0 R2 expression beadchip), while GSE49541 and GSE63067 utilized the GPL570 platform (HG-U133_Plus_2 Affymetrix Human Genome U133 Plus 2.0 Array). Supplementary Table 1 presented detailed information about these datasets. To create a comprehensive dataset, GSE49541, GSE89632, and GSE63067 were merged. Data normalization was then performed using the ComBat function from the R package sva [20] for batch correction. The data was further normalized using the normalizeBetweenArrays function from the R package limma[ [6]]. This process resulted in a Combined dataset, which incorporated 72 NAFLD samples from GSE49541, 39 NAFLD samples, and 24 control samples from GSE89632, along with 11 NAFLD samples and 7 control samples from GSE63067, totaling 122 NAFLD samples and 31 control samples.

**Fig. 1:**
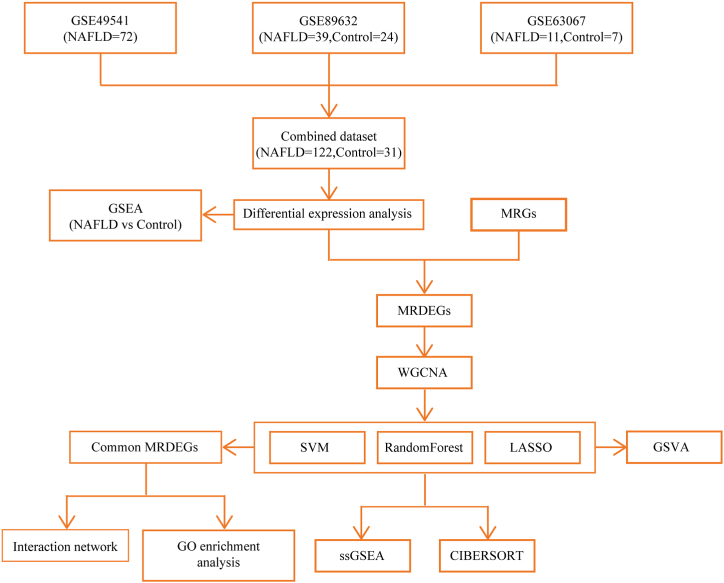
**The flowchart of the study.** NAFLD, Nonalcoholic fatty liver disease; GSEA, Gene Set Enrichment Analysis; MRGs, Mitophagy related genes; MRDEGs, Mitophagy related differentially expressed genes; WGCNA, weighted correlation network analysis; SVM, Support Vector Machine; LASSO, least absolute shrinkage and selection operator; GO, Gene Ontology; GSVA, Gene Set Variation Analysis; ssGSEA, single-sample gene-set enrichment analysis.

MRGs were sourced from the GeneCards database [21](https://www.genecards.org/). After filtering for protein-coding genes, 1620 MRGs with a relevance score >1 were retrieved using the keyword "Mitophagy." The detailed information about these MRGs is presented in Supplementary Table 2. A flow diagram of comprehensive analysis of MRDEGs was shown in Fig. 1.

### 2.2 Gene set enrichment analysis (GSEA) [22]

GSEA is commonly utilized to assess changes in pathway and biological process activities within expression data samples. Unlike focusing solely on individual gene expression alterations, GSEA takes into account the expression patterns of entire gene sets, enabling a comprehensive analysis of the functional regulatory features within gene expression data. All genes in the Combined dataset were categorized based on positive and negative logFC values. Enrichment analysis, utilizing the clusterProfiler package in R, employed parameters as seed of 2022, number of calculations of 1000, minimum number of genes contained in each gene set of 10 and maximum number of genes contained in each gene set of 500, and P-value correction method of Benjamini-Hochberg (BH). We acquired the gene set "c2.cp.all.v2022.1.Hs.symbols.gmt [All Canonical Pathways](3050)" from the Molecular Signatures Database (MSigDB) [23]. Significantly enriched pathways were identified with p.adj <0.05 and FDR (q.value) < 0.05.

### 2.3 Differentially expressed genes analysis

Differentially expressed genes between the NAFLD group samples and the Control group samples in the Combined dataset were identified using the limma package in R. The specific statistical test method used to identify differentially expressed genes was a linear model with empirical Bayesian moderation, and the method employed for multiple testing correction was the Benjamini-Hochberg procedure. Criteria for selection were set at |logFC| > 0.5 and adjusted p-value (p.adj) < 0.05. Volcano plots, generated with the ggplot2 R package, visually represent the results. The intersection of MRGs with DEGs yielded MRDEGs.

### 2.4 Weighted correlation network analysis (WGCNA)

WGCNA is a systems biology approach used to characterize gene correlation patterns across different samples. It can identify gene sets that undergo highly coordinated changes and, based on the interconnectedness within gene sets and their associations with phenotypes, identify candidate biomarker genes or therapeutic targets. WGCNA R package [24] was utilized for our analysis. The genes were sorted in descending order by standard deviation (sd) between disease controls to select the top 50%. Subsequently, the top 3000 genes with the largest absolute median difference (mad) were analyzed using WGCNA. The minimum number of modular genes was set to 40, the softpower was set to 10 (optimal softpower), and the module merge cut height was set to 0.20. The study aimed to objectively measure the correlation of various modules between the NAFLD and Control groups in the Combined dataset. The genes located in each module were identified as its signature genes. To identify all genes highly correlated with NAFLD, modules with P < 0.05 were chosen. The genes within the module, obtained after screening, intersected with MRDEGs to derive the final MRDEGs.

### 2.5 Construction and Evaluation of Diagnostic prediction model based on hub MRGs

Initially, logistic regression analysis was conducted on MRDEGs to screen those meeting the criterion of a p-value <0.05. Logistic regression is an algorithm suitable for binary classification problems, known for its high computational efficiency and strong interpretability. The expression of MRDEGs included in the logistic regression model was visually presented using a Forest Plot. Only genes meeting the logistic diagnostic model's screening criteria were used in the subsequent diagnostic model construction for MRDEGs. Hub genes were identified by combining the results of three algorithms.

Using the expression matrix and grouping information (NAFLD/Control) of the Combined dataset, the SVM algorithm [25] was employed to construct the SVM model, screening MRDEGs based on the highest accuracy and lowest error rate. Random Forest (RF) [26] a machine learning algorithm, was also used to construct the model by integrating the expression of MRDEGs in the Combined dataset's expression matrix.

Subsequently, LASSO([27]) regression analyses were performed on MRDEGs filtered by the logistic regression model using the R package glmnet [28]. To prevent overfitting, the run period was set to 500. Diagnostic model plots and variable trajectory plots were employed to visualize the outcomes of the LASSO regression analysis.

The intersection of MRDEGs contained in the LASSO model, SVM model, and Random Forest model was determined, and a Venn diagram was plotted to obtain the Hub MRGs. Combining the coefficients of Hub MRGs in the LASSO model with the expression in the Combined dataset yielded the diagnostic prediction model of Hub MRGs.

$$\text{risk}Score=\sum_{i}Coefficient\;\left({hub\;gene}_{i}\right)*mRNA\;Expression\;\left(hub\;{gene}_{i}\right)$$

Logistic regression analyses based on the expression of Hub MRGs in the Combined dataset were performed using the R package rms, and a nomogram was plotted to display the results. DCA([29]) was employed to assess the accuracy and discriminative capabilities of the diagnostic prediction model of Hub MRGs, utilizing the R package ggDCA to plot DCA graphs based on the risk scores. ROC curves were generated for the RiskScore and Hub MRGs using the R package pROC, and AUC calculations were performed.

### 2.6 Gene functional enrichment analysis

Gene Ontology (GO) [30] analysis, a standard method for large-scale functional enrichment studies encompassing biological process (BP), molecular function (MF), and cellular component (CC), was executed for Hub MRGs. The R package clusterProfile [31] was employed, applying entry screening criteria of p-value <0.05 and Benjamini-Hochberg (BH) correction.

### 2.7 Construction of mRNA-TF, mRNA-RBP, mRNA-drug, and mRNA-miRNA interaction networks

Transcription factors (TFs) binding to Hub MRGs were identified using the CHIPBase database (version 3.0, https://rna.sysu.edu.cn/chipbase/) [32] with a criterion of interactions having a Number of samples found (upstream)≥5. The resulting mRNA-TF network was visualized using Cytoscape software.

ENCORI[33]database (https://starbase.sysu.edu.cn/) was utilized to predict RNA binding proteins (RBPs) interacting with Hub MRGs, selecting interaction pairs based on a clipExpNum >5 criterion. The mRNA-RBP interaction network was visualized in Cytoscape.

The CTD[34](Comparative Toxicogenomics Database, http://ctdbase.org/) predicted drugs or small molecules interacting with Hub MRGs. Interaction pairs were screened using "Reference Count" > 1, and the resulting interaction network was visualized using Cytoscape.

ENCORI database was also used to predict miRNAs interacting with Hub MRGs. Pairs of interactions recorded at least 5 times were retained, and the mRNA-miRNA interaction network was mapped in Cytoscape.

### 2.8 Gene Set Variation Analysis (GSVA) [35]

To conduct GSVA analysis, we utilized the reference gene set "c2.cp.all.v2022.1.Hs.symbols.gmt" from the MSigDB (https://www.gsea-msigdb.org/) on the gene expression matrix of High and Low Risk groups of NAFLD samples in the Combined dataset, sorted by the RiskScore model. This facilitated the assessment of functional disparities in enriched pathways between the groups. The top 10 pathways with the highest and lowest log2FC values were selected for further analysis, ensuring p.adj <0.05.

### 2.9 Analysis of immune cell infiltration

The quantification of relative immune cell abundance was accomplished using the ssGSEA algorithm. Each immune cell type, including CD8^+^ T cells, dendritic cells, macrophages, regulatory T cells, and various other human immune cell subtypes, was labeled. The ssGSEA algorithm in the R package GSVA[35] was employed to assess the enrichment scores of samples in the Combined dataset, representing the infiltration levels of diverse immune cell types. Boxplots exhibited the infiltration abundance of immune cells between High and Low Risk groups, while Spearman's algorithm analyzed correlations among immune cells and between immune cells and Hub MRGs, visually represented through correlation dot plots.

CIBERSORT[36] an algorithm for immune infiltration analysis, was also utilized. It employed linear support vector regression to estimate the composition and abundance of immune cells in cell mixtures. The expression matrix data of the Combined dataset was uploaded to CIBERSORT and combined with the LM22 signature gene matrix. A boxplot illustrated the differences in immune cell infiltration abundance between High and Low Risk groups, and Spearman's algorithm calculated the correlation between immune cells in the two groups. Results were visualized using the R package ggplot2, and correlation dot plots depicted the correlation between immune cells and Hub MRGs.

### 2.10 Statistical analysis

All data processing and analyses were conducted using R software (Version 4.1.2). Statistical significance for normally distributed variables in comparisons between two continuous variable groups was estimated using the independent Student t-test, while the Mann-Whitney *U* test was employed for intergroup comparisons involving non-normally distributed variables. Spearman's rank correlation analysis was utilized for correlation analyses. All p-values were two-sided, with p < 0.05 considered statistically significant.

## 3 Results

### 3.1 Data processing

**Fig. 2:**
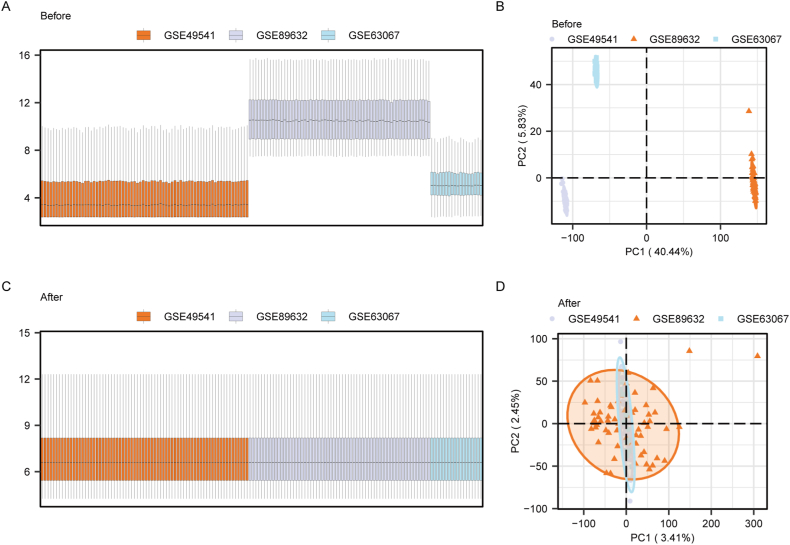
**Data processing.** A: Boxplot of Combined dataset before batch effect removal. B: Principal component analysis of Combined dataset before normalization. Each dataset was represented by a different color: orange squares for GSE49541, blue squares for GSE89632, and brown triangles for GSE63067. C: Boxplot of Combined dataset after batch effect removal. D: Principal component analysis of Combined dataset after normalization. The three datasets, GSE49541, GSE89632, and GSE63067, remained consistent with their respective color identifiers. However, the distribution of data points was tighter, with no apparent offset, indicating enhanced comparability among the datasets post-standardization. Notably, ellipses in the figure represent the density of data point distribution, where denser ellipses signify higher sample clustering. (For interpretation of the references to color in this figure legend, the reader is referred to the Web version of this article.)

A total of 3 datasets (GSE49541, GSE89632, and GSE63067) were merged, and data debatching was carried out using the ComBat function of the sva package. Normalization was performed using the normalizeBetweenArrays function of the limma package. This process yielded a Combined dataset comprising 122 NAFLD samples and 31 control samples. Box plots and principal component analysis (PCA) plots of the Combined dataset were generated both before (Fig. 2A and B) and after (Fig. 2C and D) processing according to the sample source. Our observations revealed a convergence in the expression patterns across samples in the Combined dataset and a tighter distribution of data points. This suggested that standardization procedures have enhanced comparability among the data, with batch effects being effectively mitigated during post-processing. Subsequent analyses on the Combined dataset were conducted exclusively on the batch-corrected samples.

### 3.2 GSEA for combined dataset

**Fig. 3:**
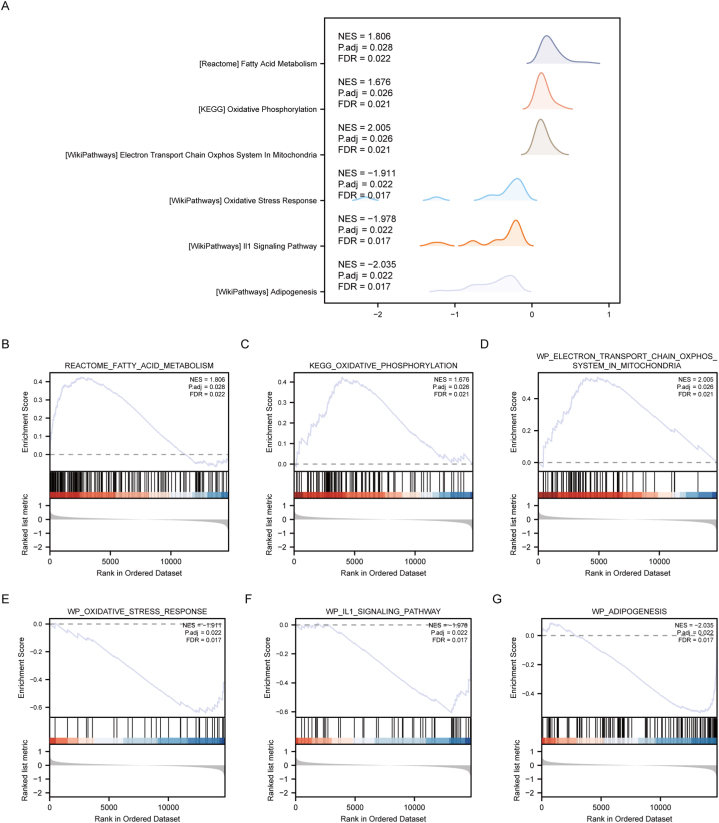
**GSEA analysis of Combined dataset.** A: Mountain map of significant enrichment terms. B–F: The results of GSEA (each term). B: REACTOME_FATTY_ACID_METABOLISM. This pathway involves the metabolic processes of fatty acids within organisms. GSEA of this pathway demonstrated significant enrichment of genes related to fatty acids in the samples, implying an enhanced activity in the fatty acid metabolism pathway. C: KEGG_OXIDATIVE_PHOSPHORYLATION. This pathway is associated with the process of cellular energy production through mitochondrial oxidative phosphorylation. Enrichment analysis revealed significant enrichment of genes related to oxidative phosphorylation in the samples, indicating a higher activity of oxidative phosphorylation within the cells. D: WP_ELECTRON_TRANSPORT_CHAIN_OXPHOS_SYSTEM_IN_MITOCHONDRIA: This is related to the oxidative phosphorylation system responsible for energy production within the mitochondria. Enrichment results indicated significant enrichment of these genes in the samples, suggesting an enhanced activity of the oxidative phosphorylation pathway within the mitochondria. E: WP_OXIDATIVE_STRESS_RESPONSE: This pathway involves the cellular mechanisms responding to oxidative stress. Enrichment analysis showed enrichment of stress-related genes in the samples, suggesting that the cells may be under oxidative stress. F: WP_IL1_SIGNALING_PATHWAY: This pathway involves the IL-1 signaling transduction pathway. Enrichment results indicated a negative enrichment of genes related to signal transduction in the samples, implying a potential downregulation of the IL1 signaling pathway in the analyzed context. G: WP_ADIPOGENESIS: This pathway involves the processes of adipocyte differentiation and development. GSEA demonstrated enrichment of genes related to adipogenesis in the samples, indicating potential changes in the signaling pathways involved in adipocyte development and differentiation. NAFLD, Nonalcoholic fatty liver disease; GSEA, Gene Set Enrichment Analysis.

GSEA was performed to analyze the association between the expression of all genes and biological processes, cellular components, and molecular functions in the NAFLD and Control groups of the Combined dataset. Screening criteria for significant enrichment were set at P.adj <0.05 and FDR value (q.value) < 0.05. The significant results were presented through a mountain map (Fig. 3A), with observed gene enrichment in pathways such as REACTOME_FATTY_ACID_METABOLISM (Fig. 3B), KEGG_OXIDATIVE_PHOSPHORYLATION (Fig. 3C), WP_ELECTRON_TRANSPORT_CHAIN_OXPHOS_SYSTEM_IN_MITOCHONDRIA (Fig. 3D), WP_OXIDATIVE_STRESS_RESPONSE (Fig. 3E), WP_IL1_SIGNALING_PATHWAY (Fig. 3F), and WP_ADIPOGENESIS (Fig. 3G) (detailed pathway information presented in Supplementary Table 3).

### 3.3 Differentially expressed genes analysis

**Fig. 4:**
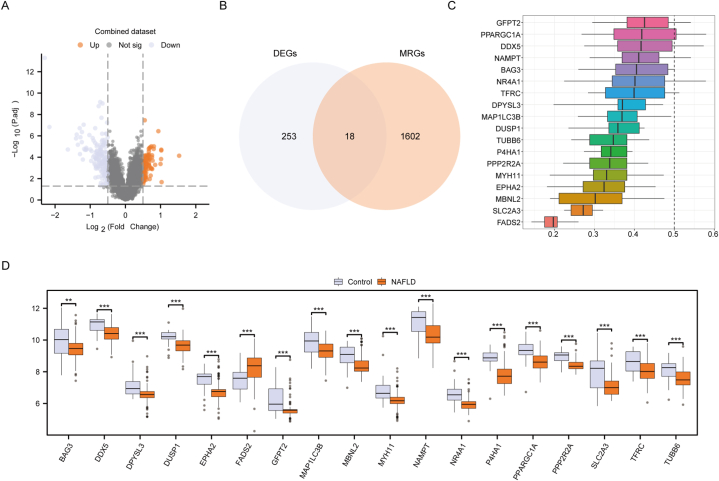
**Mitophagy-related differentially expressed genes analysis.** A: Volcano plot of DEGs between NAFLD and control group of Combined dataset. B: Venn diagram of DEGs with MRGs. C: Boxplot of the functional similarity analysis results of MRDEGs. D: Boxplot of MRDEGs between NAFLD and control group of Combined dataset. NAFLD, Nonalcoholic fatty liver disease; DEGs, Differentially expressed genes; MRGs, Mitophagy-related genes; MRDEGs, Mitophagy-related differentially expressed genes. MAP1LC3B: Microtubule-associated protein 1 light chain 3 beta; TFRC: Transferrin receptor; FADS2: Fatty acid desaturase 2; TUBB6: Tubulin beta 6 class V; NAMPT: Nicotinamide phosphoribosyltransferase; GFPT2: Glutamine--fructose-6-phosphate transaminase 2; NR4A1: Nuclear receptor subfamily 4 group A member 1; MYH11: Myosin-11; EPHA2: Ephrin type-A receptor 2; PPARGC1A: Peroxisome proliferator-activated receptor gamma coactivator 1-alpha; DDX5: DEAD-box helicase 5; BAG3: BCL2-associated athanogene 3; SLC2A3: Solute carrier family 2 member 3; PPP2R2A: Protein phosphatase 2 regulatory subunit B alpha; P4HA1: Prolyl 4-hydroxylase subunit alpha 1; DPYSL3: Dihydropyrimidinase-like 3; MBNL2: Muscleblind-like protein 2; DUSP1: Dual specificity protein phosphatase 1. ns, P ≥ 0.05; *P < 0.05; **P < 0.01; ***P < 0.001.

GSEA takes into account the expression patterns of all genes. To screen for biologically significant hub genes, we first conducted differentially expressed genes (DEG) analysis. The limma package was employed to conduct DEG analysis between the NAFLD and Control groups of the Combined dataset. A total of 271 genes met the criteria of |logFC| > 0.5 and p.adj <0.05. Within this threshold, 82 genes were highly expressed (up-regulated) in the NAFLD group compared to the Control group, while 189 genes were low-expressed (down-regulated) compared to the Control group (Fig. 4A). By intersecting the 271 DEGs with 1620 MRGs, we identified 18 MRDEGs, namely MAP1LC3B, TFRC, FADS2, TUBB6, NAMPT, GFPT2, NR4A1, MYH11, EPHA2, PPARGC1A, DDX5, BAG3, SLC2A3, PPP2R2A, P4HA1, DPYSL3, MBNL2, and DUSP1 (Fig. 4B).

The functional similarity of the 18 MRDEGs was analyzed using the R package GOSemSim, computing semantic similarity between Gene Ontology (GO) terms, sets of GO terms, gene products and gene clusters. The analysis focused on pathways in biological processes (BP), molecular function (MF), and cellular component (CC). A box plot visualized the functional similarity results (Fig. 4C), highlighting GFPT2 with the highest functional similarity among the MRDEGs.

Expression differences and trends of the 18 MRDEGs between the NAFLD group and Control group were displayed in a box plot (Fig. 4D). Significant differences were observed for all 18 MRDEGs, with FADS2 significantly upregulated in the NAFLD group and MAP1LC3B, TFRC, TUBB6, NAMPT, GFPT2, NR4A1, MYH11, EPHA2, PPARGC1A, DDX5, BAG3, SLC2A3, PPP2R2A, P4HA1, DPYSL3, MBNL2, and DUSP1 exhibiting significant down-regulation in NAFLD samples compared to the Control group.

### 3.4 WGCNA for combined dataset

**Fig. 5:**
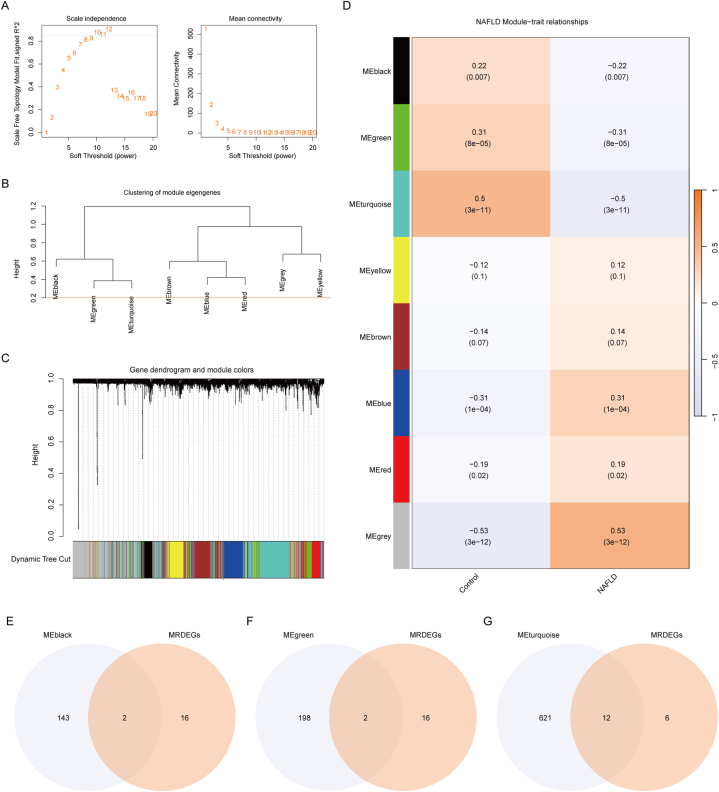
**Weighted gene co-expression network analysis (WGCNA).** A: An analysis of the scale-free fit index and the mean connectivity for selected soft threshold powers (β). B: Presentation of aggregated results for the genes module of the Combined dataset. C: Hierarchical Cluster Tree reveals co-expression modules. D: Heatmap showing the relationship between gene modules and groups. E–G: Venn diagram of MRDEGs with genes of Meblack (E), MEgreen (F), and Meturquoise (G) module. NAFLD, Nonalcoholic fatty liver disease; MRDEGs, Mitophagy related differentially expressed genes.

To further identify which differential genes play a central role in NAFLD, we conducted WGCNA analysis. WGCNA was conducted on all genes in the Combined dataset to identify co-expression modules. Initially, scale-free co-expression networks were constructed using WGCNA. The screening criterion was then set to 0.85 to determine the optimal softpower and the determined optimal soft power was 10 (Fig. 5A). Prior to merging any modules with a lower merging cut height threshold (Fig. 5B), we adjusted the module merge cut height to 0.2. Subsequently, we clustered the genes in the Combined dataset and visualized the relationship between these genes and their corresponding modules (Fig. 5C). Eight modules (MEblack, MEgreen, MEturquoise, MEyellow, MEbrown, MEblue, MEred, MEgrey) were derived based on the expression patterns of module genes and the grouping information in the Combined dataset. The correlations between these modules and phenotypes were depicted in Fig. 5D. Significance was assessed at a level of p < 0.05 for seven modules, excluding the "MEgrey" module, which was deemed non-contributory. We then identified the intersection of genes in these modules with 18 MRDEGs (Fig. 5E–G). Following this procedure, the gene list was ultimately narrowed down to 16 MRDEGs: P4HA1, FADS2, NAMPT, MAP1LC3B, BAG3, SLC2A3, PPARGC1A, TUBB6, EPHA2, DDX5, MBNL2, DUSP1, PPP2R2A, NR4A1, MYH11, and DPYSL3.

### 3.5 Construction of Diagnostic Prediction Model Based on Hub MRGs

**Fig. 6:**
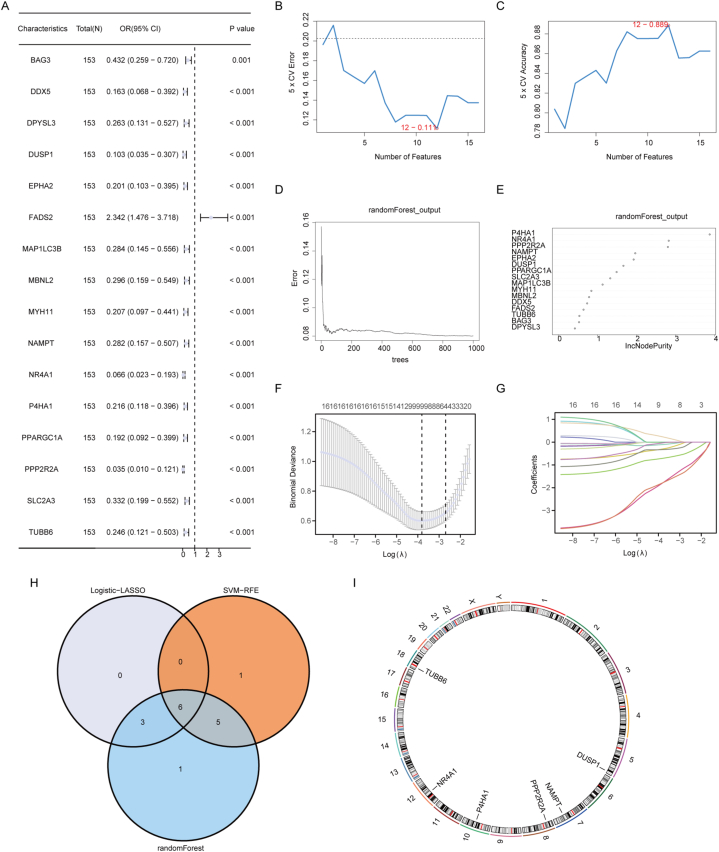
**Construction of Diagnostic Prediction Model Based on Hub MRGs.** A: Forest Plot of logistic regression of MRDEGs. B–C: The curve of change in the predicted error and true value of each gene in SVM algorithm. D: Plot of the model training error for the Random Forest algorithm. E: Random Forest model showing MRDEGs (listed in IncNodePurity descending order). F: Cross-validation curves in LASSO logistic regression algorithm. G: Regression coefficient path diagram of LASSO logistic regression algorithm. H: Venn diagram of MRDEGs in the three algorithms. I: Chromosomal localization plots for Hub MRGs. NAFLD, Nonalcoholic fatty liver disease; MRDEGs, Mitophagy related differentially expressed genes; SVM, Support Vector Machine; LASSO, Least Absolute Shrinkage and Selection Operator; MRGs, Mitophagy-related genes. NR4A1: Nuclear receptor subfamily 4 group A member 1; PPP2R2A: Protein phosphatase 2 regulatory subunit B alpha; P4HA1: Prolyl 4-hydroxylase subunit alpha 1; TUBB6: Tubulin beta 6 class V; DUSP1: Dual specificity protein phosphatase 1; NAMPT: Nicotinamide phosphoribosyltransferase.

To develop the diagnostic prediction model for NAFLD based on Hub MRGs, we initially conducted logistic regression using the expression levels of the aforementioned 16 MRDEGs and the grouping information (NAFLD/Control) of the Combined dataset samples. The MRDEGs were screened with a P-value threshold of less than 0.05. All 16 MRDEGs were incorporated into the final logistic regression model, and the expression of these MRDEGs was visualized in a Forest Plot (Fig. 6A).

Subsequently, SVM models were constructed based on the 16 MRDEGs using the SVM algorithm to identify genes with the lowest error rate (Fig. 6B) and the highest accuracy (Fig. 6C). Our findings revealed that the SVM model achieved optimal accuracy when considering 12 genes, specifically NR4A1, PPP2R2A, P4HA1, MYH11, DPYSL3, PPARGC1A, TUBB6, SLC2A3, MAP1LC3B, DUSP1, EPHA2, and NAMPT.

The Random Forest algorithm was also employed to analyze the expression of the 16 MRDEGs in the Combined dataset (Fig. 6D). Only IncNodePurity values greater than 0.5 were considered for targeted analysis results. The random forest algorithm identified 15 diagnostic markers (Fig. 6E), including P4HA1, NR4A1, PPP2R2A, NAMPT, EPHA2, DUSP1, PPARGC1A, SLC2A3, MAP1LC3B, MYH11, MBNL2, DDX5, FADS2, TUBB6, and BAG3.

Subsequently, we constructed a diagnostic model based on the expressions of the 16 MRDEGs in the Combined dataset using LASSO regression analysis. The results were visualized through cross-validation curves (Fig. 6F) and regression coefficient path diagram (Fig. 6G). The LASSO regression model comprised nine MRDEGs: DDX5, DUSP1, FADS2, MBNL2, NAMPT, NR4A1, P4HA1, PPP2R2A, and TUBB6.

To identify Hub MRGs, we determined the intersection of MRDEGs obtained through LASSO, SVM, and Random Forest models. Six Hub MRGs were identified and displayed in the Venn diagram (Fig. 6H): NR4A1, PPP2R2A, P4HA1, TUBB6, DUSP1, and NAMPT.

Then we combined the coefficients of Hub MRGs in the LASSO model and the expression in Combined dataset to derive the diagnostic prediction model of the six Hub MRGs.

RiskScore=NR4A1*−1.793929928+PPP2R2A*−1.78725378+P4HA1*−0.709501842+TUBB6*−0.033928631+DUSP1*−0.326923522+NAMPT*−0.009948108

Furthermore, the RCircos package in R was employed to positionally annotate and map the chromosomal localization of the six Hub MRGs (Fig. 6I), providing insight into the specific distribution of these MRGs on the chromosome.

### 3.6 Evaluation of Diagnostic prediction model

**Fig. 7:**
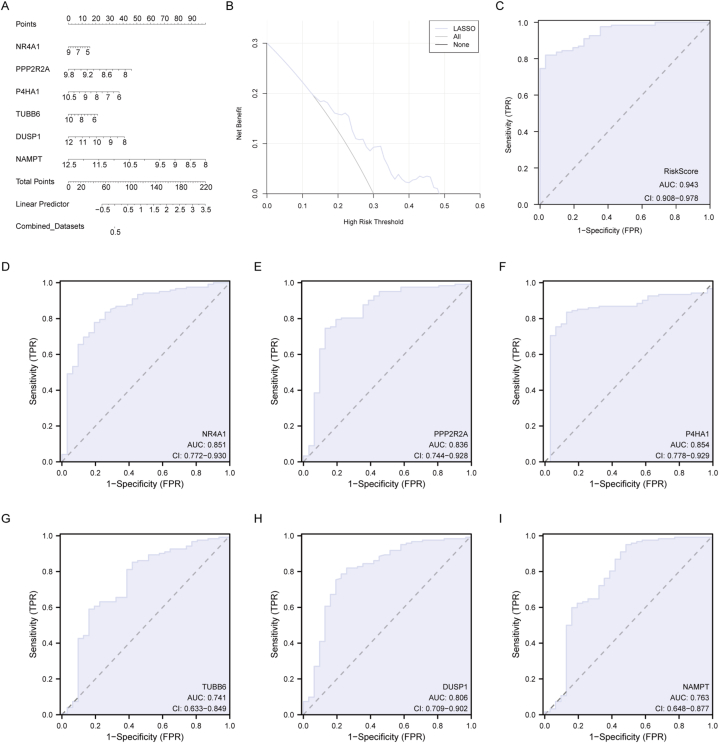
**Evaluation of Diagnostic prediction Model.** A: Nomogram of the diagnostic prediction model. B: DCA of the diagnostic prediction model. C: ROC curves of diagnostic prediction model. D–I: ROC curve for the six genes in the diagnostic prediction model. DCA: Decision curve analysis; ROC, receiver operating characteristic curve; AUC, Area Under the Curve.

To further validate the MRGs diagnostic prediction model, we developed a binary logistic regression model by analyzing the expression of the six Hub MRGs within the Combined dataset. The rms package in R was then utilized to construct a Nomogram illustrating the contribution of these six MRGs to the logistic regression model (Fig. 7A). The results highlighted that NAMPT expression among the Hub MRGs was particularly influential in the logistic regression model. Subsequently, we employed DCA to evaluate the clinical utility of the Hub MRGs diagnostic prediction model (Fig. 7B). The DCA plot indicated that the model's effectiveness improves over a range when the line consistently remains above the lines for "All positive" and "All negative." The results underscored the model's accuracy in diagnosing the occurrence of NAFLD. ROC curves were generated based on the Riskscore model and the grouping information (NAFLD/Control) of the Combined dataset. As depicted in Fig. 7C, the diagnostic prediction model exhibited high accuracy for identifying NAFLD occurrence, with an AUC of 0.943. Furthermore, individual diagnostic ROC curves for the six Hub MRGs were plotted (Fig. 7D–I). The results revealed that NR4A1 (Fig. 7D, AUC = 0.851), PPP2R2A (Fig. 7E, AUC = 0.836), P4HA1 (Fig. 7F, AUC = 0.854), TUBB6 (Fig. 7G, AUC = 0.741), DUSP1 (Fig. 7H, AUC = 0.806), and NAMPT (Fig. 7I, AUC = 0.763) each demonstrated a certain degree of accuracy in diagnosing NAFLD occurrence, suggesting that these six genes have the potential to be diagnostic biomarkers of NAFLD.

### 3.7 GO enrichment analysis of hub MRGs

**Fig. 8:**
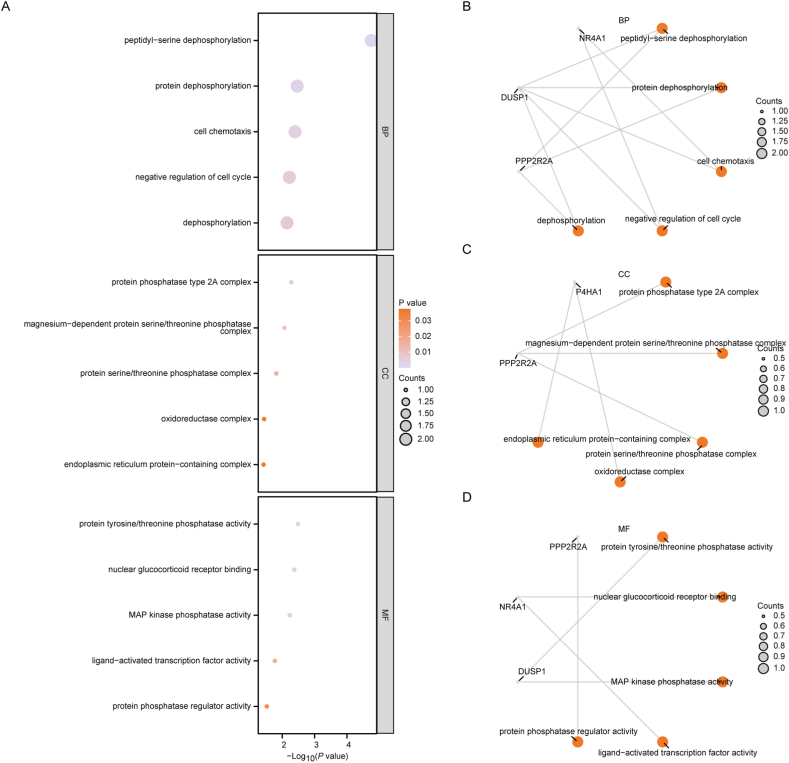
**GO enrichment analysis of Hub MRGs.** A: Bubble plot of enriched GO terms. B–D: Cnetplots of BP pathway (B), CC pathway (C), and MF pathway (D) in the GO enrichment analysis of Hub MRGs. Purple dots represent specific genes and orange dots represent specific pathways in the Cnetplot. MRGs, Mitophagy-related genes; GO, Gene Ontology; BP, biological process; CC, cellular component; MF, molecular function. Annotation of the GO terms in figure: Peptidyl-serine dephosphorylation – Refers to the removal of a phosphate group from serine residues on peptides. Protein dephosphorylation – The process of removing phosphate groups from proteins, which is important for regulating protein function. Cell chemotaxis – Movement of a cell or organism in response to a chemical stimulus. Negative regulation of cell cycle – Processes that stop or slow down the cell cycle, preventing the cell from dividing. Dephosphorylation – The general process of removing phosphate groups from molecules like proteins. Protein phosphatase type 2A complex – A type of enzyme complex involved in catalyzing the removal of phosphate groups. Magnesium-dependent protein serine/threonine phosphatase complex – A group of enzymes requiring magnesium ions to catalyze the dephosphorylation of serine/threonine residues in proteins. Protein serine/threonine phosphatase complex – Enzymes that are specialized in removing phosphate groups from serine/threonine residues in proteins. Oxidoreductase complex – Complexes of enzymes involved in oxidation-reduction reactions, transferring electrons from one molecule to another. Endoplasmic reticulum protein-containing complex – Proteins or complexes involving proteins that reside in the endoplasmic reticulum. Protein tyrosine/threonine phosphatase activity – Enzymatic activity involved in removing phosphate from tyrosine and threonine residues in proteins. Nuclear glucocorticoid receptor binding – Interaction with the glucocorticoid receptor located within the nucleus, often with a regulatory role. MAP kinase phosphatase activity – Enzymes that deactivate MAP kinases by dephosphorylation, thus regulating various cellular processes. Ligand-activated transcription factor activity – Transcription factors that are activated by the binding of specific ligands to influence gene expression. Protein phosphatase regulator activity – Enzymatic regulation involving modulating the activity of protein phosphatases. (For interpretation of the references to color in this figure legend, the reader is referred to the Web version of this article.)

To elucidate the potential functional relationships of the Hub MRGs, we conducted Gene Ontology (GO) enrichment analysis, employing a screening criterion for enriched entries with a p-value less than 0.05. Pathways meeting this criterion were considered statistically significant. The outcomes revealed that the six Hub MRGs participated in various Biological Processes (BPs), with a predominant involvement in peptidyl-serine dephosphorylation, protein dephosphorylation, cell chemotaxis, negative regulation of the cell cycle, and dephosphorylation. Molecular Functions (MFs) primarily encompassed protein tyrosine/threonine phosphatase activity, nuclear glucocorticoid receptor binding, MAP kinase phosphatase activity, ligand-activated transcription factor activity, and protein phosphatase regulator activity. Regarding Cellular Components (CC), the main components included the protein phosphatase type 2A complex, magnesium-dependent protein serine/threonine phosphatase complex, protein serine/threonine phosphatase complex, oxidoreductase complex, and endoplasmic reticulum protein-containing complex. Detailed pathway information was presented in Supplementary Table 4. The results of the GO function enrichment analysis were visually represented using a bubble diagram (Fig. 8A). Furthermore, the enrichment outcomes for BP (Fig. 8B), CC (Fig. 8C), and MF (Fig. 8D) were depicted in the form of a cnetplot.

### 3.8 Construction of mRNA-TF, mRNA-RBP, mRNA-drug, and mRNA-miRNA interaction networks for hub MRGs

**Fig. 9:**
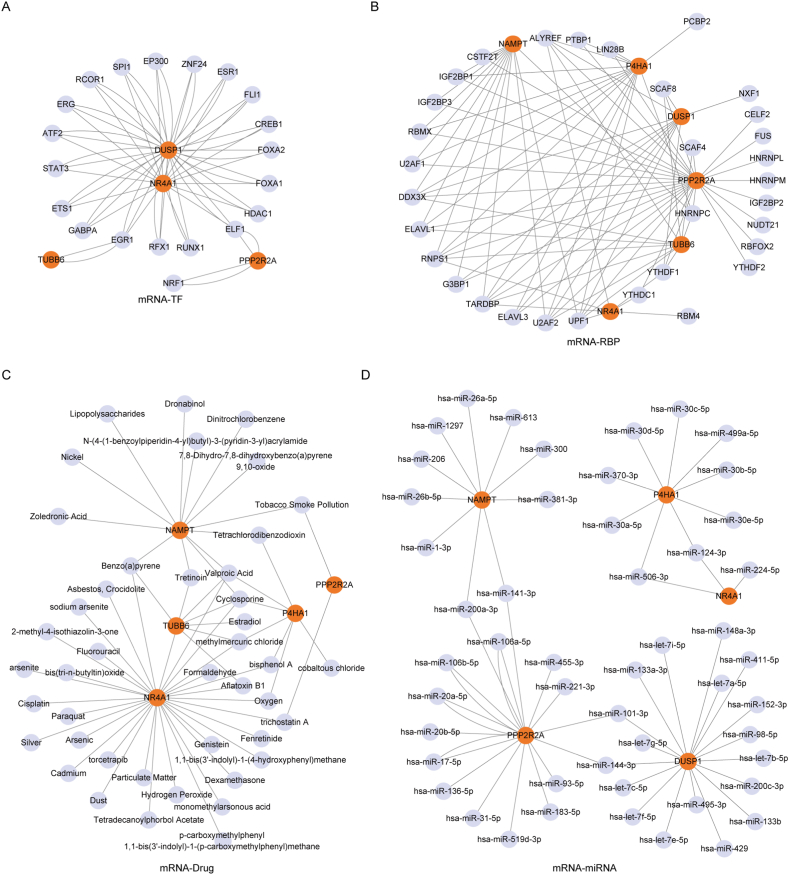
**mRNA-TF, mRNA-RBP, mRNA-drug, and mRNA-miRNA interaction networks for Hub MRGs.** A: mRNA-TF network of Hub MRGs. Orange circles represented mRNA nodes, while gray circles represented transcription factors (TFs). Solid lines depicted the interactions between mRNA and transcription factors. B: mRNA-RBP network of Hub MRGs. Orange circles represented mRNA nodes, and gray circles represented RNA-binding proteins (RBPs). Solid lines indicated interactions between mRNA and RNA-binding proteins. C: mRNA-drug network of Hub MRGs. Orange circles represented biomolecules influenced by chemical substances, and gray circles with black text represented various chemical substances. Solid lines represented the interactions between specific chemical substances and biomolecules. D: mRNA-miRNA network of Hub MRGs. Orange circles represented mRNA molecules, while gray circles represented microRNAs (miRNAs) regulating mRNA. Solid lines denoted interactions between mRNA and miRNA. MRGs, Mitophagy-related genes; TF, Transcription factors; RBP, RNA binding protein. (For interpretation of the references to color in this figure legend, the reader is referred to the Web version of this article.)

To delve into the regulatory mechanisms of the Hub MRGs, we initiated a search in the CHIPBase (version 3.0) database to identify transcription factors binding to the six Hub MRGs (NR4A1, PPP2R2A, P4HA1, TUBB6, DUSP1, NAMPT). Subsequent screening revealed a total of 41 pairs of interaction data between four Hub MRGs (NR4A1, DUSP1, PPP2R2A, TUBB6) and 20 transcription factors (TFs). Visualization of this interaction network was performed using Cytoscape software (Fig. 9A). Detailed mRNA-TF interactions were presented in Supplementary Table 5.

We utilized the ENCORI database to predict the interaction between RBPs and the six Hub MRGs. This analysis resulted in a total of 86 pairs of mRNA-RBP interactions, encompassing the aforementioned 6 MRGs and 32 RBP molecules (Fig. 9B). Detailed mRNA-RBP interactions were provided in Supplementary Table 6.

For the anticipation of potential medicinal drugs or small molecule compounds interacting with the 6 Hub MRGs, we turned to the CTD database. Ultimately, we obtained a total of 60 pairs of mRNA-drug interactions, involving 5 Hub MRGs (NR4A1, NAMPT, P4HA1, PPP2R2A, and TUBB6), and 43 drug molecules (Fig. 9C). Detailed mRNA-drug interactions were presented in Supplementary Table 7.

Lastly, we employed the ENCORI database to predict miRNAs interacting with the 6 Hub MRGs. Following screening, we identified a total of 56 pairs of mRNA-miRNA interactions, comprising 5 Hub MRGs (DUSP1, NAMPT, NR4A1, P4HA1, and PPP2R2A), and 50 miRNA molecules (Fig. 9D). Detailed mRNA-miRNA interactions are outlined in Supplementary Table 8.

### 3.9 GSVA between High and Low Risk group of NAFLD samples

**Fig. 10:**
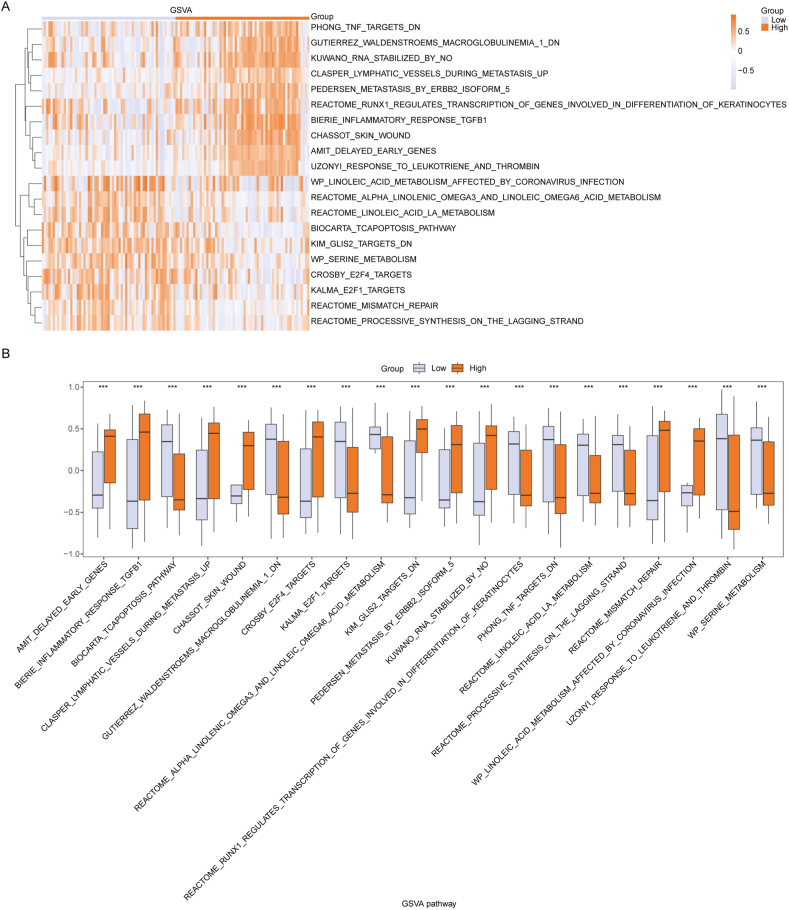
**GSVA analysis between High and Low-Risk group.** A: Heatmap of the results of the GSVA enrichment analysis of genes between High and Low-Risk group in the Combined dataset. The vertical axis of the heatmap listed different pathway or gene set names. Horizontally, there are two groups representing experimental subgroups, namely, High and Low-risk group of NAFLD samples. The depth of color represents the enrichment level of gene sets in the corresponding subgroup. Colors range from orange to yellow-white, where orange indicates positive enrichment scores, suggesting upregulation or enhanced activity of the associated pathway in that subgroup; while yellow-white represents scores close to zero or negative, indicating downregulation or no significant change in the pathway. B: Boxplot of the GSVA enrichment pathways between High and Low-Risk group in the Combined dataset. The box in the boxplot represents the interquartile range of the data (the middle 50% of the data), with the median represented by the middle line. Whiskers indicate the range of data variation. Orange and gray boxes represent the High and Low-Risk group, respectively. GSVA, Gene Set Variation Analysis. ns, P ≥ 0.05; *P < 0.05; **P < 0.01; ***P < 0.001. (For interpretation of the references to color in this figure legend, the reader is referred to the Web version of this article.)

To better assess NAFLD, we categorized the NAFLD samples to High and Low-risk group based on their median RiskScore. To examine the biological behavioral differences between the High and Low-Risk group of NAFLD samples, all genes within the NAFLD samples of the Combined dataset were subjected to Gene Set Variation Analysis (GSVA). We selected the top 10 pathways with the highest and lowest log2FC values in the p.adj<0.05 of the GSVA enrichment findings for further examination, resulting in a total of 20 pathways (details provided in Supplementary Table 9). The differential expression of these 20 pathways between High and Low-Risk group was visualized using heatmaps generated with the R package pheatmap (Fig. 10A). The results showed that pathways such as TNF targets DN, Waldenstroems macroglobulinemia 1 DN, RNA stabilized by NO, Lymphatic vessels during metastasis up, Metastasis by ERBB2 isoform 5, RUNX1 regulates transcription of genes involved in differentiation of keratinocytes, Inflammatory response TGFB1, Skin wound, Delayed early genes and Response to leukotriene and thrombin were highly active in the High-Risk group. However, Linoleic acid metabolism affected by coronavirus infection, Alpha linolenic omega3 and linoleic omega6 acid metabolism, Linoleic acid la metabolism, Tcapoptosis pathway, GLIS2 targets DN, Serine metabolism, E2F4 targets, E2F1 targets, Mismatch repair and Processive synthesis on the lagging strand pathways exhibited heightened activity in the Low-Risk Group. Additionally, the Mann-Whitney *U* test was applied to assess the variation in the 20 pathways between High and Low-Risk group, and the results were presented through a box plot (Fig. 10B), revealing statistically significant differences in the expression of all 20 pathways in NAFLD samples from the Combined dataset.

### 3.10 Immune cell infiltration analysis between High and Low Risk groups by ssGSEA

**Fig. 11:**
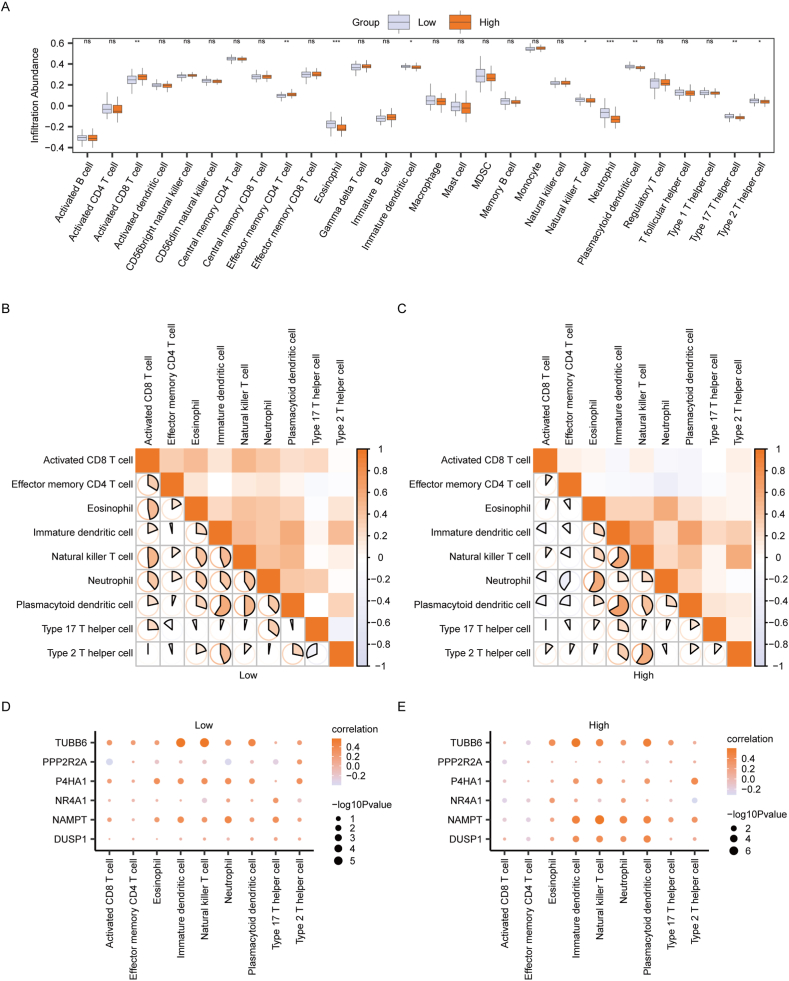
**Immune infiltration analysis between High and Low-Risk group by ssGSEA.** A: Differences in infiltration abundance of 28 immune cells between High and Low-Risk group. Orange and gray boxes represent the High and Low-Risk group, respectively. B–C: Correlation between different immune cells in Low-risk group(B) and High-risk group(C). Color: Ranging from orange to white, representing the values of correlation coefficients. Orange indicated positive correlation (values close to +1), white indicated no correlation (values close to 0), and gray indicated negative correlation (values close to −1). The size and fill of the shapes corresponded to the logarithmic transformation of the -log10 P-values (larger shapes represented lower P-values, indicating higher statistical significance) D–E: Correlation between six hub MRGs and infiltration of immune cells in Low-risk group(D) and High-risk group(E). Circle Size represented the logarithmic transformation of the -log10 P-value, where larger circles corresponded to lower P-values, indicating stronger statistical significance. Color: Ranging from orange to white, representing correlation coefficients. Orange indicated positive correlation, while white indicated negative correlation. ssGSEA, single-sample gene-set enrichment Analysis; MRGs, Mitophagy-related genes. ns, P ≥ 0.05; *P < 0.05; **P < 0.01; ***P < 0.001. (For interpretation of the references to color in this figure legend, the reader is referred to the Web version of this article.)

To investigate differences in immune infiltration between the High and Low-Risk groups in the Combined dataset NAFLD samples, we utilized the ssGSEA algorithm to calculate the abundance of 28 immune cells in both groups. Subsequently, we analyzed the degree of infiltration difference between the group using the Mann-Whitney *U* test and illustrated the results through a box plot (Fig. 11A). Significantly different infiltration abundances of 9 immune cells in the Combined dataset NAFLD samples were identified between the High and Low-Risk group (P < 0.05). These immune cells included Activated CD8 T cell, Effector memory CD4 T cell, Eosinophil, Immature dendritic cell, Natural killer T cell, Neutrophil, Plasmacytoid dendritic cell, Type 17 T helper cell, and Type 2 T helper cell. We further computed the correlations between the infiltration abundances of these nine immune cells. In the Low-Risk group, correlations between immune cells were predominantly positive, with the strongest correlations observed between Plasmacytoid dendritic cell and Immature dendritic cell (Fig. 11B). Similarly, in the High-Risk group, correlations between immune cells were predominantly positive, with Plasmacytoid dendritic cells and Immature dendritic cells exhibiting the strongest correlations (Fig. 11C).

Additionally, we investigated the correlation between the abundance of the nine immune cell infiltrates and the expression of the six Hub MRGs in NAFLD samples from both High and Low-Risk group of the Combined dataset. In the Low-Risk group, a greater number of positive correlations between immune cells and Hub MRGs. The most significant correlation was found between Immature dendritic cells and TUBB6 (Fig. 11D). Similarly, in the High-Risk group, a substantial number of positive correlations between immune cells and Hub MRGs were identified, with the most robust correlation observed between Natural killer T cells and NAMPT (Fig. 11E).

### 3.11 Immune cell infiltration analysis between High and Low Risk group by CIBERSOR

**Fig. 12:**
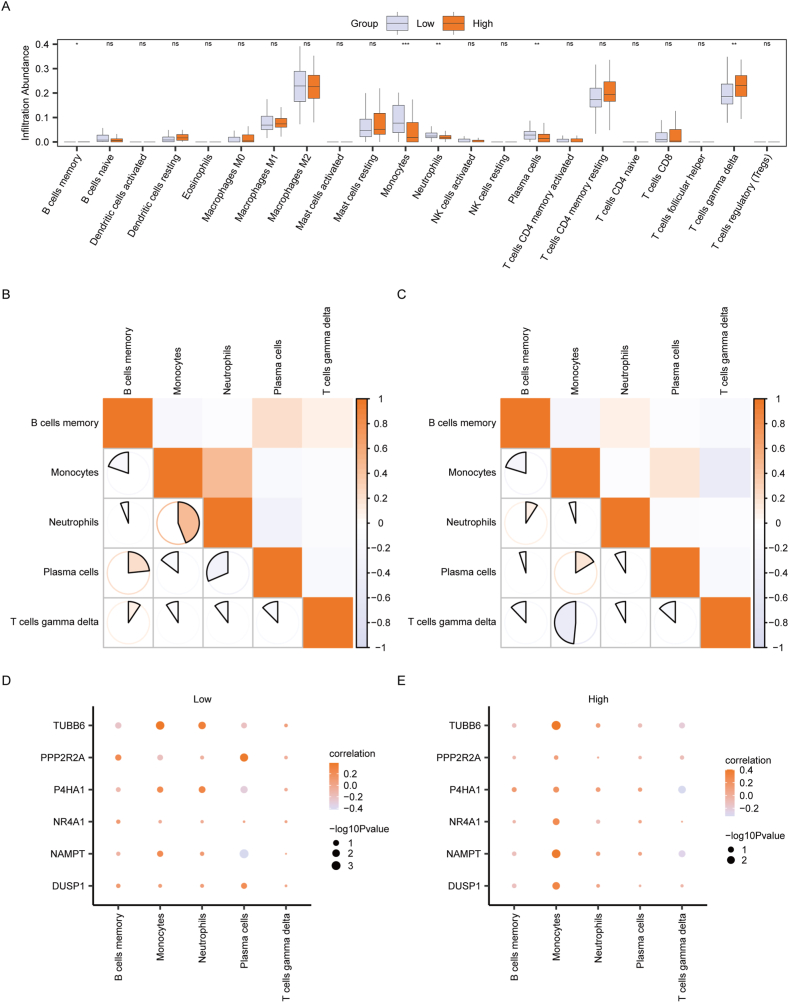
**Immune infiltration analysis between High and Low-Risk group by CIBERSORT.** A: Differences in infiltration abundance of 22 immune cells between High and Low-Risk group. Orange and gray boxes represent the High and Low-Risk group, respectively. B–C: Correlation between different immune cells in Low-risk group(B) and High-risk group(C). Color: Ranging from orange to white, representing the values of correlation coefficients. Orange indicated positive correlation (values close to +1), white indicated no correlation (values close to 0), and gray indicated negative correlation (values close to −1). The size and fill of the shapes corresponded to the logarithmic transformation of the -log10 P-values (larger shapes represented lower P-values, indicating higher statistical significance) D–E: Correlation between six hub MRGs and infiltration of immune cells in Low-risk group(D) and High-risk group(E). Circle Size represented the logarithmic transformation of the -log10 P-value, where larger circles corresponded to lower P-values, indicating stronger statistical significance. Color: Ranging from orange to white, representing correlation coefficients. Orange indicated positive correlation, while white indicated negative correlation.MRGs, Mitophagy-related genes. ns, P ≥ 0.05; *P < 0.05; **P < 0.01; ***P < 0.001. (For interpretation of the references to color in this figure legend, the reader is referred to the Web version of this article.)

Subsequently, we employed the CIBERSORT algorithm to compute the infiltration abundance of 22 immune cells in the Low and High-Risk group. The Mann-Whitney *U* test was utilized to examine the degree of variance in the infiltration of the 22 immune cells between the High and Low-Risk group, and the results were presented in a box plot (Fig. 12A). A significant difference in the infiltration abundance of five immune cells between the High and Low-Risk group was observed. These immune cells included B cells memory, Monocytes, Neutrophils, Plasma cells, and T cells gamma delta. Using the "spearman" algorithm, we determined the correlation between the abundance of these five immune cells in both the Low and High-Risk groups of NAFLD samples. In the Low-Risk group, the highest correlation was observed between Neutrophils and Monocytes (Fig. 12B), while in the High-Risk group, the strongest correlation was found between Monocytes and T cells gamma delta (Fig. 12C). Furthermore, we calculated the correlation between the abundance of these five immune cells and the expression of the six Hub MRGs in patient samples from both the Low and High-Risk groups in the Combined dataset. The findings were illustrated in correlation dot plots, which revealed a nearly equal distribution of positive and negative correlations between immune cells and Hub MRGs in the Low-Risk group. Notably, the most robust correlation was identified between plasma cells and NAMPT (Fig. 12D). Likewise, in the High-Risk group, we observed a balanced distribution of positive and negative correlations between immune cells and Hub MRGs, with Monocytes and TUBB6 exhibiting the most pronounced correlation (Fig. 12E).

## 4 Discussion

NAFLD is a prevalent cause of chronic liver disease globally, and it is strongly associated with metabolic disorders, such as obesity, insulin resistance, hyperlipidemia, and hyperglycemia. Consequently, an international expert group led by Eslam et al. proposed renaming NAFLD to Metabolic Dysfunction-Associated Fatty Liver Disease (MAFLD) [37]. The current gold standard for diagnosing NAFLD remains hepatic puncture biopsy, despite its invasiveness, limited applicability, and other restrictions. Clinical and imaging methods face challenges in achieving early and dependable diagnosis. Mitochondria, which play a crucial role in cellular metabolic pathways, are thought to be a critical factor in the development of NAFLD. Mitophagy has a significant role in NAFLD due to the selective degradation of damaged mitochondria through autophagy [38]. Therefore, targeting mitophagy for NAFLD holds great potential for clinical applications. Previous studies have established a link between mitophagy and NAFLD, highlighting the importance of mitochondrial metabolism in the disease's pathogenesis. However, the reciprocal interaction between mitophagy and immune infiltration in NAFLD progression remains elusive.

This study examined the function of MRGs in NAFLD, identified a set of six Hub MRGs with biomarker capability for NAFLD for the first time, and constructed a diagnostic prediction model based on these six Hub MRGs. These Hub MRGs and the diagnostic prediction model exhibited excellent predictive capabilities. According to our investigation, this study is the first to combine mitophagy in NAFLD with DEGs, establishing and evaluating a diagnostic prediction model that could serve as an adjunct for NAFLD diagnosis. Additionally, we explored the relationship between immune infiltration in NAFLD and Hub MRGs, as well as the regulatory network of MRGs, providing a more comprehensive and integrated perspective of the role of mitophagy in NAFLD.

Previous research has established the critical roles of lipid metabolism and mitochondrial function in NAFLD[[39], [40]]. In our study, we combined three datasets (GSE49541, GSE89632, and GSE63067) and performed GSEA. The results confirmed enrichment pathways related to lipid metabolism and mitochondrial function, consistent with previous studies. Furthermore, the IL-1 signaling pathway, explicitly associated with NAFLD[41], was also enriched, supporting the reliability of following investigations using the combined dataset. Through the intersection of DEGs and MRGs, we identified 18 MRDEGs. Subsequently, through WGCNA, logistic regression, SVM, RF, and Lasso regression, we identified six Hub MRGs (NR4A1, PPP2R2A, P4HA1, TUBB6, DUSP1, NAMPT), all significantly downregulated in NAFLD samples compared to the control group.

Nuclear receptor subfamily 4 group A member 1 (NR4A1), also known as Nur77, TR3, or NGFI-B, is a member of the metabolic nuclear receptor family expressed in energy metabolism tissues such as the liver, and is associated with metabolic diseases [42]. Zhou et al. [43] suggested that NR4A1 is significantly upregulated in high-fat diet-induced NAFLD, further activating the DNA-PKcs/p53 pathway, leading to mitochondrial dysfunction, inhibiting mitophagy, and promoting NAFLD progression. Melatonin can reverse this process, maintaining mitochondrial homeostasis in NAFLD. Nevertheless, this contradicts our findings of downregulated NR4A1 expression in our study. This discrepancy could be attributed to several factors that differ between our study and previous research. Firstly, the sample characteristics such as the genetic background, age, sex, and health status of the subjects may vary, potentially influencing NR4A1 expression. Secondly, analytical methods such as the sensitivity and specificity of the assays used to measure gene expression might account for the observed differences. Furthermore, our research was a secondary analysis of existing data, which lacked direct experimental validation. On the other hand, Liang et al. [44] reported that depletion of NR4A1 exacerbated Homocysteine-induced NAFLD, suggesting a protective role of NR4A1 against NAFLD. The inconsistencies between these studies and ours may also stem from the variations in the NAFLD models employed and the methods for inducing NR4A1 loss or gain-of-function. These discrepancies highlight the need for further experimental investigations to elucidate the precise mechanisms underlying NR4A1-mediated effects in NAFLD.

Protein phosphatase 2A (PP2A) complex, composed of catalytic subunit C, structural subunit A, and variable regulatory subunit B, regulates various cellular processes by dephosphorylating numerous cellular proteins. The diversity of the four regulatory B subunit families, including PPP2R2A, determines the substrate specificity and subcellular localization of PP2A[45]. Mutations in the PPP2R2A gene have been associated with diabetes and insulin resistance, participating in the regulation of glucose metabolism [46]. Li et al. [47] indicated that PPP2R2A was involved in regulating Leydig cells' mitochondrial function, thereby impacting testosterone secretion in hu sheep. Our study uncovered a robust association between PPP2R2A and NAFLD. However, there are currently no reported mechanisms linking PPP2R2A to the occurrence and development of NAFLD.

P4HA1 encodes a component of proline 4-hydroxylase, a key enzyme in collagen synthesis, which is associated with liver fibrosis and hepatocellular carcinoma [48]. Previous studies have demonstrated a significant association between P4HA1 and NAFLD[[49], [50]], consistent with our findings of decreased expression levels in NAFLD. Tubulin beta 6 class V (TUBB6), one of eight beta-tubulin isotypes (class I-VI), is distributed in various human cells. TUBB6 has been linked to cancer invasion and metastasis in aggressive malignancies [51]. Mitochondria can adjust their intracellular distribution through interactions with the cellular cytoskeleton, adapting to cellular energy and metabolic demands. This interaction is crucial for mitochondrial renewal and removal, and abnormalities in this process contribute to the development of NAFLD[52]. Mitochondrial transfer also occurs between cells, and disruption of intercellular mitochondrial transfer promotes NAFLD development [53]. We hypothesize that the downregulation of TUBB6 expression in NAFLD may regulate NAFLD progression through this mechanism.

DUSP1, also known as MKP 1 (mitogen-activated protein kinase phosphatase 1), is a regulatory molecule in the MAPK pathway, highly expressed in the heart and liver. Various studies have exhibited the contribution of DUSP1 in regulating the injury of cardiomyocytes by regulating mitophagy [54,55]. Similar to our findings, Zhang et al. [56] showed that DUSP1 was downregulated in NAFLD, participating in the regulation of NAFLD through modulated MAPK activity. Nicotinamide phosphoribosyltransferase (NAMPT) is a regulator of the intracellular nicotinamide adenine dinucleotide (NAD) pool, present in almost all cells. Its NAD biosynthetic activity can regulate cell metabolism and mitochondrial biogenesis, influencing inflammation, oxidative stress, and protein toxicity [57]. Previous studies have demonstrated the regulatory role of NAMPT in NAFLD, and upregulating NAMPT through various strategies has been shown to prevent steatosis and inflammation [58,59]. NAMPT has emerged as a potential therapeutic target for NAFLD.

Based on the constructed RiskScore model, we stratified NAFLD patients into high-risk and low-risk groups. GSVA analysis revealed distinct biological behavior characteristics between the high-risk and low-risk groups. In the pathogenesis of NAFLD, various immune cell types play distinct roles in liver inflammation and disease progression [11]. Kupffer cells, as liver-resident macrophages, initiate defense against pathogens and toxins by producing pro-inflammatory cytokines such as TNF-α and IL-1β. CD8^+^ T cells promote liver inflammation and injury, while Th17 cells exacerbate hepatic inflammation, leading to liver damage. Regulatory T cells (Tregs), whose levels decrease in NAFLD, may compromise the regulation of liver inflammation. Moreover, inflammatory mediators produced by activated B cells can aggravate liver inflammation and injury. Natural killer (NK) cells, involved in the innate immune response, contribute to liver inflammation, further worsening hepatic damage. Natural killer T (NKT) cells, acting as a bridge between innate and adaptive immunity, can be activated in NAFLD, intensifying liver inflammation through cytokine production and interaction with other immune cells. Understanding the roles of these immune cell types is crucial for comprehending the intricate immune response in NAFLD and its impact on liver pathology. Notably, our ssGSEA and CIBERSORT algorithms revealed differences in immune infiltration abundance between high and low-risk group, further supporting the varied immune cell infiltration in NAFLD. These cells exhibit intricate cross-talk, collectively regulating the onset and development of NAFLD.

The ssGSEA algorithm indicated that plasmacytoid dendritic cells and immature dendritic cells exhibited the strongest correlation in both high-risk and low-risk groups. Meanwhile, the CIBERSORT algorithm revealed that neutrophils and monocytes had the strongest correlation in the low-risk group, whereas monocytes and T cells gamma delta showed the strongest correlation in the high-risk group. This suggested a promising direction for future research on the immune interactions in NAFLD. Additionally, both algorithms collectively highlight the potential key role of neutrophils. Previous studies have shown that neutrophils, one of the first leukocytes activated after Kupffer cell recruitment in NAFLD, can secrete large amounts of quantities of IL-6 and proteases, including myeloperoxidase, neutrophil elastase, and protease 3, enhancing the inflammatory environment, promoting ROS, and causing hepatocyte damage, thus contributing to the progression of NASLD[[60], [61], [62], [63], [64]]. On the other hand, neutrophils effectively reduced hepatic inflammation and fibrosis in various animal models, and depletion of neutrophils resulted in a general aggravation of hepatic inflammation [65,66], aligning with our study's finding of lower infiltration of neutrophils in the high-risk group.

Moreover, recent research indicates that mitophagy also plays a regulatory role in the immune system, suggesting a potential crosstalk between these processes in the coordinated regulation of NAFLD progression [14,67]. Consequently, we conducted a further analysis of the correlation between the six Hub MRGs and infiltrating immune cells. The results revealed a significant correlation between almost all of these 6 Hub MRGs and immune cells, with TUBB6 and NAMPT exhibiting a particularly strong association. Specifically, the ssGSEA correlation analysis highlighted a robust positive correlation between TUBB6 and Immature dendritic cells in the low-risk group, while in the high-risk group, NAMPT demonstrated the strongest positive correlation with Natural killer T cells. Similarly, CIBERSORT analysis showcased a significant negative correlation between NAMPT and Plasma cells in the low-risk group, whereas TUBB6 exhibited the strongest positive correlation with Monocytes in the high-risk group. These findings further substantiate the crucial roles of mitophagy and the immune system in the occurrence and development of NAFLD, deepening our understanding of the MRG-dependent immune status and microenvironment in NAFLD. Future research should delve into the molecular mechanisms underlying the intricate interactions between these genes and immune cells.

This study, focusing on mitophagy, employed various bioinformatics approaches to explore Hub MRGs, diagnostic prediction model, and related immunopathological mechanisms associated with NAFLD. Nevertheless, our study possesses some limitations. Firstly, our study was retrospective in nature and relied on the secondary analysis of existing datasets, which may introduce inherent biases and limitations associated with retrospective analyses. Additionally, gene expression data provides a snapshot of the transcriptional state of a tissue at a given time point, but does not capture post-transcriptional or post-translational modifications that may be important for gene function. Secondly, confounding factors such as age, sex, ethnicity, or comorbidities that may influence gene expression and immune infiltration patterns in NAFLD. Thirdly, the lack of external datasets and experimental validation poses a limitation to the robustness and generalizability of our prediction models. We recognize the importance of validating our findings in independent cohorts and experimental settings to strengthen the reliability of our results. Fourthly, the immune infiltration analyses were limited to a subset of immune cell types and did not capture the full complexity of the immune microenvironment in NAFLD. Furthermore, while our study sheds light on the potential role of mitophagy in NAFLD pathogenesis, further mechanistic studies are warranted to elucidate the causal relationships between mitophagy dysregulation, immune regulation, and the development and progression of NAFLD.

In the future, we aim to integrate additional data from NAFLD patients and control groups, ensuring diversity and representativeness in the dataset. Advanced algorithms and optimized models will be employed for this purpose. Model validation and cross-validation will be conducted in independent cohorts to assess stability. Furthermore, independent validation across multiple medical institutions will be considered to ensure the model's generalizability and reliability. Statistical methods will be applied to comprehensively evaluate the accuracy, sensitivity, specificity, and other metrics of the model. The interpretation of model predictions will be provided, exploring their potential application in clinical practice. Comparative analysis and integration with existing clinical assessment tools will be performed to produce a comprehensive assessment report, consolidating multiple evaluation metrics. Based on the predictive model results and information from clinical assessment tools, a standardized diagnostic workflow will be designed to assist physicians in diagnosing the onset and progression of NAFLD more rapidly and accurately. Moreover, personalized treatment plans will be devised for patients based on comprehensive assessment results, enabling precise monitoring and treatment tailored to individual characteristics and risk levels.

## 5 Conclusion

Through bioinformatics approaches, we identified six Hub-MRGs in NAFLD with biomarker capability and constructed a diagnostic prediction model based on these six genes. The assessment of the prediction model demonstrated excellent diagnostic performance, rendering it suitable for NAFLD diagnosis. Furthermore, immune infiltration and correlation analysis revealed that these six mitophagy-related genes may regulate the occurrence and progression of NAFLD by modulating immune responses. This study also delved into the regulatory networks of these key genes. Looking ahead, our research findings pave the way for several promising avenues in NAFLD research. Specifically, these identified hub MRGs could serve as targets for validation in prospective clinical studies aimed at validating their utility as diagnostic biomarkers. Moreover, understanding the intricate regulatory networks of these genes opens doors for targeted therapeutic interventions. By elucidating how these genes modulate immune responses, novel therapeutic strategies could be developed to halt or reverse the progression of NAFLD. In essence, our study not only enhances our understanding of NAFLD but also provides tangible directions for future clinical and basic research efforts, ultimately contributing to the advancement of diagnostic and therapeutic approaches in managing this complex disease.

## Data availability statement

The datasets used for analysis in this study (GSE49541, GSE89632 and GSE63067) were derived from the GEO database (https://www.ncbi.nlm.nih.gov/geo/).

## CRediT authorship contribution statement

**Zhenguo Luo:** Writing – original draft, Visualization, Software, Formal analysis, Data curation. **Shu Yan:** Writing – original draft, Visualization, Validation, Formal analysis, Data curation. **Yu Chao:** Writing – original draft, Supervision, Investigation. **Ming Shen:** Writing – review & editing, Writing – original draft, Visualization, Supervision, Methodology, Formal analysis, Data curation, Conceptualization.

## Declaration of competing interest

The authors declare that they have no known competing financial interests or personal relationships that could have appeared to influence the work reported in this paper.
