# Supplementary material for: Unveiling the mitophagy puzzle in non-alcoholic fatty liver disease (NAFLD): Six hub genes for early diagnosis and immune modulatory roles
Source: Heliyon. 2024 Mar 31;10(7):e28935. doi: 10.1016/j.heliyon.2024.e28935 (PMC11004814; doi:10.1016/j.heliyon.2024.e28935)
Supplement: Multimedia component 1 [file mmc1.docx]

### Table 1. NAFLD Dataset Information list.

|  | GSE49541 | GSE89632 | GSE63067 |
| --- | --- | --- | --- |
| Platform | GPL570 | GPL14951 | GPL570 |
| Species | Homo sapiens | Homo sapiens | Homo sapiens |
| Experiment type | Expression profiling by array | Expression profiling by array | Expression profiling by array |
| Tissue | liver | liver | liver |
| Samples in Control group | 0 | 24 | 7 |
| Samples in NAFLD group | 72 | 39 | 11 |
| Reference | (18) | (19) | (20) |

NAFLD：Nonalcoholic fatty liver disease.
