# Supplementary material for: Unveiling the mitophagy puzzle in non-alcoholic fatty liver disease (NAFLD): Six hub genes for early diagnosis and immune modulatory roles
Source: Heliyon. 2024 Mar 31;10(7):e28935. doi: 10.1016/j.heliyon.2024.e28935 (PMC11004814; doi:10.1016/j.heliyon.2024.e28935)
Supplement: Multimedia component 3 [file mmc3.docx]

**Table 3. GSEA enrichment analysis results of Combined dataset Control-NAFLD group genes.**

| ID | setSize | enrichmentScore | NES | p.adjust | qvalue |
| --- | --- | --- | --- | --- | --- |
| WP_ADIPOGENESIS | 126 | -0.53198 | -2.03541 | 0.02175 | 0.01694 |
| WP_IL1_SIGNALING_PATHWAY | 48 | -0.60676 | -1.97777 | 0.02175 | 0.01694 |
| WP_OXIDATIVE_STRESS_RESPONSE | 33 | -0.6392 | -1.91137 | 0.02175 | 0.01694 |
| WP_ELECTRON_TRANSPORT_CHAIN_OXPHOS_SYSTEM_IN_MITOCHONDRIA | 67 | 0.53462 | 2.004931 | 0.02645 | 0.0206 |
| KEGG_OXIDATIVE_PHOSPHORYLATION | 93 | 0.423447 | 1.675556 | 0.026485 | 0.020628 |
| REACTOME_FATTY_ACID_METABOLISM | 153 | 0.424824 | 1.80552 | 0.028046 | 0.021844 |

GSEA：Gene Set Enrichment Analysis; NAFLD：Nonalcoholic fatty liver disease.
