# Supplementary material for: Unveiling the mitophagy puzzle in non-alcoholic fatty liver disease (NAFLD): Six hub genes for early diagnosis and immune modulatory roles
Source: Heliyon. 2024 Mar 31;10(7):e28935. doi: 10.1016/j.heliyon.2024.e28935 (PMC11004814; doi:10.1016/j.heliyon.2024.e28935)
Supplement: Multimedia component 4 [file mmc4.docx]

**Table 4. GO enrichment analysis results.**

| ONTOLOGY | ID | Description | GeneRatio | BgRatio | pvalue |
| --- | --- | --- | --- | --- | --- |
| BP | GO:0070262 | peptidyl-serine dephosphorylation | 2/6 | 21/18800 | 1.78E-05 |
| BP | GO:0006470 | protein dephosphorylation | 2/6 | 291/18800 | 0.003437 |
| BP | GO:0060326 | cell chemotaxis | 2/6 | 315/18800 | 0.004015 |
| BP | GO:0045786 | negative regulation of cell cycle | 2/6 | 387/18800 | 0.006002 |
| BP | GO:0016311 | dephosphorylation | 2/6 | 422/18800 | 0.007102 |
| CC | GO:0000159 | protein phosphatase type 2A complex | 1/6 | 17/19594 | 0.005195 |
| CC | GO:0005963 | magnesium-dependent protein serine/threonine phosphatase complex | 1/6 | 28/19594 | 0.008545 |
| CC | GO:0008287 | protein serine/threonine phosphatase complex | 1/6 | 50/19594 | 0.015215 |
| CC | GO:1990204 | oxidoreductase complex | 1/6 | 120/19594 | 0.036192 |
| CC | GO:0140534 | endoplasmic reticulum protein-containing complex | 1/6 | 125/19594 | 0.037676 |
| MF | GO:0008330 | protein tyrosine/threonine phosphatase activity | 1/6 | 10/18410 | 0.003255 |
| MF | GO:0035259 | nuclear glucocorticoid receptor binding | 1/6 | 13/18410 | 0.00423 |
| MF | GO:0033549 | MAP kinase phosphatase activity | 1/6 | 18/18410 | 0.005853 |
| MF | GO:0098531 | ligand-activated transcription factor activity | 1/6 | 52/18410 | 0.01683 |
| MF | GO:0019888 | protein phosphatase regulator activity | 1/6 | 93/18410 | 0.029933 |

GO：Gene Ontology; BP，biological process; CC：cellular component.
