# Supplementary material for: Unveiling the mitophagy puzzle in non-alcoholic fatty liver disease (NAFLD): Six hub genes for early diagnosis and immune modulatory roles
Source: Heliyon. 2024 Mar 31;10(7):e28935. doi: 10.1016/j.heliyon.2024.e28935 (PMC11004814; doi:10.1016/j.heliyon.2024.e28935)
Supplement: Multimedia component 5 [file mmc5.docx]

### Table 5. mRNA-TF interaction network nodes.

| mRNA | TF |  | mRNA | TF |  | mRNA | TF |
| --- | --- | --- | --- | --- | --- | --- | --- |
| DUSP1 | ATF2 |  | DUSP1 | RFX1 |  | NR4A1 | FOXA1 |
| DUSP1 | CREB1 |  | DUSP1 | RUNX1 |  | NR4A1 | FOXA2 |
| DUSP1 | EGR1 |  | DUSP1 | SPI1 |  | NR4A1 | GABPA |
| DUSP1 | ELF1 |  | DUSP1 | STAT3 |  | NR4A1 | HDAC1 |
| DUSP1 | EP300 |  | DUSP1 | ZNF24 |  | NR4A1 | RCOR1 |
| DUSP1 | ERG |  | NR4A1 | ATF2 |  | NR4A1 | RFX1 |
| DUSP1 | ESR1 |  | NR4A1 | CREB1 |  | NR4A1 | RUNX1 |
| DUSP1 | ETS1 |  | NR4A1 | EGR1 |  | NR4A1 | SPI1 |
| DUSP1 | FLI1 |  | NR4A1 | ELF1 |  | NR4A1 | STAT3 |
| DUSP1 | FOXA1 |  | NR4A1 | EP300 |  | NR4A1 | ZNF24 |
| DUSP1 | FOXA2 |  | NR4A1 | ERG |  | PPP2R2A | ELF1 |
| DUSP1 | GABPA |  | NR4A1 | ESR1 |  | PPP2R2A | NRF1 |
| DUSP1 | HDAC1 |  | NR4A1 | ETS1 |  | TUBB6 | EGR1 |
| DUSP1 | RCOR1 |  | NR4A1 | FLI1 |  |  |  |

TF：Transcription factors.
