# Supplementary material for: Unveiling the mitophagy puzzle in non-alcoholic fatty liver disease (NAFLD): Six hub genes for early diagnosis and immune modulatory roles
Source: Heliyon. 2024 Mar 31;10(7):e28935. doi: 10.1016/j.heliyon.2024.e28935 (PMC11004814; doi:10.1016/j.heliyon.2024.e28935)
Supplement: Multimedia component 6 [file mmc6.docx]

### Table 6. mRNA-RBP interaction network nodes.

| mRNA | RBP |  | mRNA | RBP |  | mRNA | RBP |
| --- | --- | --- | --- | --- | --- | --- | --- |
| DUSP1 | ALYREF |  | NR4A1 | YTHDC1 |  | PPP2R2A | IGF2BP2 |
| DUSP1 | DDX3X |  | NR4A1 | YTHDF1 |  | PPP2R2A | IGF2BP3 |
| DUSP1 | ELAVL3 |  | P4HA1 | ALYREF |  | PPP2R2A | LIN28B |
| DUSP1 | G3BP1 |  | P4HA1 | DDX3X |  | PPP2R2A | NUDT21 |
| DUSP1 | NXF1 |  | P4HA1 | ELAVL1 |  | PPP2R2A | PTBP1 |
| DUSP1 | RNPS1 |  | P4HA1 | ELAVL3 |  | PPP2R2A | RBFOX2 |
| DUSP1 | SCAF8 |  | P4HA1 | HNRNPC |  | PPP2R2A | RBMX |
| DUSP1 | TARDBP |  | P4HA1 | IGF2BP1 |  | PPP2R2A | RNPS1 |
| DUSP1 | U2AF2 |  | P4HA1 | LIN28B |  | PPP2R2A | SCAF4 |
| DUSP1 | UPF1 |  | P4HA1 | PCBP2 |  | PPP2R2A | SCAF8 |
| DUSP1 | YTHDF1 |  | P4HA1 | PTBP1 |  | PPP2R2A | TARDBP |
| NAMPT | CSTF2T |  | P4HA1 | RBMX |  | PPP2R2A | U2AF1 |
| NAMPT | DDX3X |  | P4HA1 | RNPS1 |  | PPP2R2A | U2AF2 |
| NAMPT | ELAVL1 |  | P4HA1 | TARDBP |  | PPP2R2A | UPF1 |
| NAMPT | G3BP1 |  | P4HA1 | U2AF1 |  | PPP2R2A | YTHDC1 |
| NAMPT | HNRNPC |  | P4HA1 | U2AF2 |  | PPP2R2A | YTHDF1 |
| NAMPT | IGF2BP1 |  | P4HA1 | UPF1 |  | PPP2R2A | YTHDF2 |
| NAMPT | IGF2BP3 |  | PPP2R2A | ALYREF |  | TUBB6 | ALYREF |
| NAMPT | RBMX |  | PPP2R2A | CELF2 |  | TUBB6 | DDX3X |
| NAMPT | RNPS1 |  | PPP2R2A | CSTF2T |  | TUBB6 | ELAVL1 |
| NAMPT | TARDBP |  | PPP2R2A | DDX3X |  | TUBB6 | HNRNPC |
| NAMPT | U2AF1 |  | PPP2R2A | ELAVL1 |  | TUBB6 | RNPS1 |
| NAMPT | U2AF2 |  | PPP2R2A | ELAVL3 |  | TUBB6 | SCAF4 |
| NAMPT | UPF1 |  | PPP2R2A | FUS |  | TUBB6 | SCAF8 |
| NR4A1 | ALYREF |  | PPP2R2A | G3BP1 |  | TUBB6 | TARDBP |
| NR4A1 | RBM4 |  | PPP2R2A | HNRNPC |  | TUBB6 | U2AF2 |
| NR4A1 | RNPS1 |  | PPP2R2A | HNRNPL |  | TUBB6 | UPF1 |
| NR4A1 | TARDBP |  | PPP2R2A | HNRNPM |  | TUBB6 | YTHDF1 |
| NR4A1 | UPF1 |  | PPP2R2A | IGF2BP1 |  |  |  |

RBP，RNA binding protein.
