# Supplementary material for: Unveiling the mitophagy puzzle in non-alcoholic fatty liver disease (NAFLD): Six hub genes for early diagnosis and immune modulatory roles
Source: Heliyon. 2024 Mar 31;10(7):e28935. doi: 10.1016/j.heliyon.2024.e28935 (PMC11004814; doi:10.1016/j.heliyon.2024.e28935)
Supplement: Multimedia component 7 [file mmc7.docx]

### Table 7. mRNA-drug interaction network nodes.

| mRNA | drug |  | mRNA | drug |
| --- | --- | --- | --- | --- |
| NR4A1 | 2-methyl-4-isothiazolin-3-one | | NAMPT | Lipopolysaccharides |
| NR4A1 | Aflatoxin B1 | | NAMPT | N-(4-(1-benzoylpiperidin-4-yl)butyl)-3-(pyridin-3-yl)acrylamide |
| NR4A1 | Arsenic |  | NAMPT | Nickel |
| NR4A1 | arsenite |  | NAMPT | Tetrachlorodibenzodioxin |
| NR4A1 | Asbestos, Crocidolite | | NAMPT | Tobacco Smoke Pollution |
| NR4A1 | Benzo(a)pyrene | | NAMPT | Tretinoin |
| NR4A1 | bisphenol A | | NAMPT | Valproic Acid |
| NR4A1 | Cadmium |  | NAMPT | Zoledronic Acid |
| NR4A1 | Cyclosporine | | NR4A1 | 1,1-bis(3'-indolyl)-1-(4-hydroxyphenyl)methane |
| NR4A1 | Dexamethasone | | NR4A1 | bis(tri-n-butyltin)oxide |
| NR4A1 | Dust |  | NR4A1 | Cisplatin |
| NR4A1 | Formaldehyde | | NR4A1 | Fenretinide |
| NR4A1 | Genistein |  | NR4A1 | Fluorouracil |
| NR4A1 | Hydrogen Peroxide | | NR4A1 | p-carboxymethylphenyl 1,1-bis(3'-indolyl)-1-(p-carboxymethylphenyl)methane |
| NR4A1 | methylmercuric chloride | | NR4A1 | torcetrapib |
| NR4A1 | monomethylarsonous acid | | P4HA1 | bisphenol A |
| NR4A1 | Oxygen |  | P4HA1 | cobaltous chloride |
| NR4A1 | Paraquat |  | P4HA1 | Cyclosporine |
| NR4A1 | Particulate Matter | | P4HA1 | methylmercuric chloride |
| NR4A1 | Silver |  | P4HA1 | Oxygen |
| NR4A1 | sodium arsenite | | P4HA1 | Tetrachlorodibenzodioxin |
| NR4A1 | Tetradecanoylphorbol Acetate | | P4HA1 | Valproic Acid |
| NR4A1 | Tretinoin |  | PPP2R2A | Tobacco Smoke Pollution |
| NR4A1 | trichostatin A | | PPP2R2A | trichostatin A |
| NR4A1 | Valproic Acid | | TUBB6 | Aflatoxin B1 |
| NAMPT | 7,8-Dihydro-7,8-dihydroxybenzo(a)pyrene 9,10-oxide | | TUBB6 | Benzo(a)pyrene |
| NAMPT | Benzo(a)pyrene | | TUBB6 | Cyclosporine |
| NAMPT | Cyclosporine | | TUBB6 | Estradiol |
| NAMPT | Dinitrochlorobenzene | | TUBB6 | methylmercuric chloride |
| NAMPT | Dronabinol | | TUBB6 | Valproic Acid |
