# Supplementary material for: Unveiling the mitophagy puzzle in non-alcoholic fatty liver disease (NAFLD): Six hub genes for early diagnosis and immune modulatory roles
Source: Heliyon. 2024 Mar 31;10(7):e28935. doi: 10.1016/j.heliyon.2024.e28935 (PMC11004814; doi:10.1016/j.heliyon.2024.e28935)
Supplement: Multimedia component 8 [file mmc8.docx]

### Table 8. mRNA-miRNA interaction network nodes.

| mRNA | miRNA |  | mRNA | miRNA |
| --- | --- | --- | --- | --- |
| DUSP1 | hsa-let-7a-5p | | NR4A1 | hsa-miR-224-5p |
| DUSP1 | hsa-let-7b-5p | | NR4A1 | hsa-miR-124-3p |
| DUSP1 | hsa-let-7c-5p | | NR4A1 | hsa-miR-506-3p |
| DUSP1 | hsa-let-7e-5p | | P4HA1 | hsa-miR-30a-5p |
| DUSP1 | hsa-let-7f-5p | | P4HA1 | hsa-miR-30c-5p |
| DUSP1 | hsa-miR-98-5p | | P4HA1 | hsa-miR-30d-5p |
| DUSP1 | hsa-miR-101-3p | | P4HA1 | hsa-miR-30b-5p |
| DUSP1 | hsa-miR-148a-3p | | P4HA1 | hsa-miR-124-3p |
| DUSP1 | hsa-let-7g-5p | | P4HA1 | hsa-miR-30e-5p |
| DUSP1 | hsa-let-7i-5p | | P4HA1 | hsa-miR-370-3p |
| DUSP1 | hsa-miR-133a-3p | | P4HA1 | hsa-miR-499a-5p |
| DUSP1 | hsa-miR-144-3p | | P4HA1 | hsa-miR-506-3p |
| DUSP1 | hsa-miR-152-3p | | PPP2R2A | hsa-miR-17-5p |
| DUSP1 | hsa-miR-200c-3p | | PPP2R2A | hsa-miR-20a-5p |
| DUSP1 | hsa-miR-133b | | PPP2R2A | hsa-miR-31-5p |
| DUSP1 | hsa-miR-429 | | PPP2R2A | hsa-miR-93-5p |
| DUSP1 | hsa-miR-495-3p | | PPP2R2A | hsa-miR-101-3p |
| DUSP1 | hsa-miR-411-5p | | PPP2R2A | hsa-miR-106a-5p |
| NAMPT | hsa-miR-26a-5p | | PPP2R2A | hsa-miR-183-5p |
| NAMPT | hsa-miR-26b-5p | | PPP2R2A | hsa-miR-221-3p |
| NAMPT | hsa-miR-1-3p | | PPP2R2A | hsa-miR-141-3p |
| NAMPT | hsa-miR-141-3p | | PPP2R2A | hsa-miR-144-3p |
| NAMPT | hsa-miR-206 | | PPP2R2A | hsa-miR-136-5p |
| NAMPT | hsa-miR-200a-3p | | PPP2R2A | hsa-miR-106b-5p |
| NAMPT | hsa-miR-381-3p | | PPP2R2A | hsa-miR-200a-3p |
| NAMPT | hsa-miR-613 | | PPP2R2A | hsa-miR-20b-5p |
| NAMPT | hsa-miR-300 | | PPP2R2A | hsa-miR-519d-3p |
| NAMPT | hsa-miR-1297 | | PPP2R2A | hsa-miR-455-3p |
