# Supplementary material for: Unveiling the mitophagy puzzle in non-alcoholic fatty liver disease (NAFLD): Six hub genes for early diagnosis and immune modulatory roles
Source: Heliyon. 2024 Mar 31;10(7):e28935. doi: 10.1016/j.heliyon.2024.e28935 (PMC11004814; doi:10.1016/j.heliyon.2024.e28935)
Supplement: Multimedia component 9 [file mmc9.docx]

**Table 9. GSVA analysis results of Combined dataset High-Low Riskscore group genes.**

|  | logFC | AveExpr | t | adj.P.Val | B |
| --- | --- | --- | --- | --- | --- |
| BIERIE_INFLAMMATORY_RESPONSE_TGFB1 | -0.44481 | 0.043481 | -4.68433 | 0.000676 | 3.547103 |
| AMIT_DELAYED_EARLY_GENES | -0.43019 | 0.011648 | -6.37758 | 3.89E-06 | 10.78744 |
| CHASSOT_SKIN_WOUND | -0.42312 | -0.0092 | -4.9822 | 0.000291 | 4.720057 |
| REACTOME_RUNX1_REGULATES_TRANSCRIPTION_OF_GENES_INVOLVED_IN_DIFFERENTIATION_OF_KERATINOCYTES | -0.39535 | 0.040383 | -4.31752 | 0.001698 | 2.172555 |
| PHONG_TNF_TARGETS_DN | -0.39404 | 0.03035 | -4.85835 | 0.000427 | 4.226433 |
| UZONYI_RESPONSE_TO_LEUKOTRIENE_AND_THROMBIN | -0.38768 | -0.01284 | -6.15695 | 9.55E-06 | 9.773293 |
| GUTIERREZ_WALDENSTROEMS_MACROGLOBULINEMIA_1_DN | -0.3862 | -0.03002 | -4.61406 | 0.000808 | 3.277653 |
| CLASPER_LYMPHATIC_VESSELS_DURING_METASTASIS_UP | -0.38445 | -0.00701 | -6.58528 | 2.3E-06 | 11.75812 |
| KUWANO_RNA_STABILIZED_BY_NO | -0.38065 | 0.09159 | -4.47994 | 0.001177 | 2.771312 |
| PEDERSEN_METASTASIS_BY_ERBB2_ISOFORM_5 | -0.37396 | 0.006716 | -5.36244 | 8.83E-05 | 6.285378 |
| REACTOME_PROCESSIVE_SYNTHESIS_ON_THE_LAGGING_STRAND | 0.303483 | 0.010406 | 4.553496 | 0.000971 | 3.047695 |
| REACTOME_LINOLEIC_ACID_LA_METABOLISM | 0.305645 | 0.005112 | 3.703787 | 0.007784 | 0.061776 |
| WP_SERINE_METABOLISM | 0.306 | 0.097876 | 4.450329 | 0.001269 | 2.660969 |
| REACTOME_MISMATCH_REPAIR | 0.307971 | -0.01746 | 4.900169 | 0.000379 | 4.392174 |
| KIM_GLIS2_TARGETS_DN | 0.323454 | 0.133866 | 4.55394 | 0.000971 | 3.049374 |
| WP_LINOLEIC_ACID_METABOLISM_AFFECTED_BY_CORONAVIRUS_INFECTION | 0.327728 | -0.02079 | 3.03413 | 0.030987 | -1.94145 |
| REACTOME_ALPHA_LINOLENIC_OMEGA3_AND_LINOLEIC_OMEGA6_ACID_METABOLISM | 0.328675 | 0.002708 | 4.929753 | 0.000341 | 4.509999 |
| KALMA_E2F1_TARGETS | 0.331713 | 0.01786 | 4.06865 | 0.003302 | 1.286908 |
| CROSBY_E2F4_TARGETS | 0.357208 | 0.037184 | 4.271499 | 0.001937 | 2.005843 |
| BIOCARTA_TCAPOPTOSIS_PATHWAY | 0.399722 | -0.01523 | 5.219369 | 0.000137 | 5.687842 |
